# Ligand–Metal Charge Transfer Induced *via* Adjustment of Textural Properties Controls the Performance of Single-Atom Catalysts during Photocatalytic Degradation

**DOI:** 10.1021/acsami.1c02243

**Published:** 2021-05-24

**Authors:** Jiaxu Liu, Yajun Zou, Daniel Cruz, Aleksandr Savateev, Markus Antonietti, Gianvito Vilé

**Affiliations:** † Department of Chemistry, Materials, and Chemical Engineering “Giulio Natta”, Politecnico di Milano, Piazza Leonardo da Vinci 32, Milan 20133, Italy; ‡ State Key Laboratory of Fine Chemicals, Department of Catalytic Chemistry and Engineering, Dalian University of Technology, Ganjingzi District, Linggong Road 2, Dalian 116024, China; § Department of Colloid Chemistry, Max Planck Institute of Colloids and Interfaces, Potsdam-Golm Science Park, Am Mühlenberg 1 OT Golm, Potsdam 14476, Germany; ∥ Department of Inorganic Chemistry, Fritz-Haber-Institut der Max-Planck-Gesellschaft, Faradayweg 4-6, Berlin 14195, Germany; ⊥ Department of Heterogeneous Reactions, Max Planck Institute for Chemical Energy Conversion, Mülheim an der Ruhr 45470, Germany

**Keywords:** single-atom catalysis, carbon nitride, ligand-to-metal charge transfer, catalytic materials, green chemistry

## Abstract

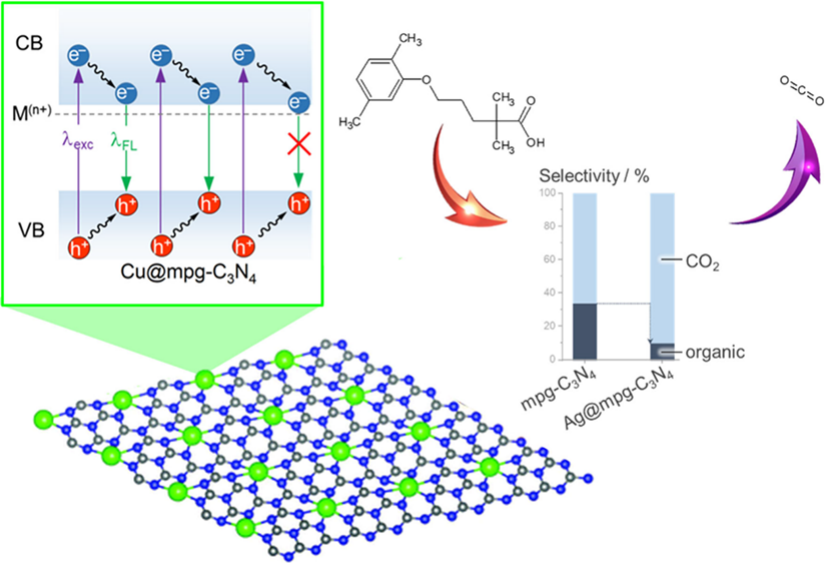

Because of their peculiar nitrogen-rich structure, carbon nitrides are convenient polydentate ligands for designing single atom-dispersed photocatalysts. However, the relation between catalysts’ textural properties and their photophysical–photocatalytic properties is rarely elaborated. Herein, we report the preparation and characterization of a series of single-atom heterogeneous catalysts featuring highly dispersed Ag and Cu species on mesoporous graphitic C_3_N_4_. We show that adjustment of materials textural properties and therefore metal single-atom coordination mode enables ligand-to-metal charge transfer (LMCT) or ligand-to-metal-to-ligand charge transfer (LMLCT), properties that were long speculated in single-atom catalysis but never observed. We employ the developed materials in the degradation of organic pollutants under irradiation with visible light. Kinetic investigations under flow conditions show that single atoms of Ag and Cu decrease the number of toxic organic fragmentation products while leading to a higher selectivity toward full degradation. The results correlate with the selected mode of charge transfer in the designed photocatalysts and provide a new understanding of how the local environment of a single-atom catalyst affects the surface structure and reactivity. The concepts can be exploited further to rationally design and optimize other single-atom materials.

## Introduction

Single-atom catalysts (SACs) have recently emerged as a new class of materials bridging the gap between the homogeneous and heterogeneous catalysis worlds.^1−3^ These materials represent the utmost utilization of precious metals while offering more facile preparation, handling, and recovery compared to traditional catalytic systems.^4,5^ Due to the unsaturated coordination of the active center as well as quantum and support effects, over the past few years, SACs were improved to show extraordinary catalytic activity and selectivity toward specific products in important transformations such as hydrogenation, oxidation, carbon–carbon coupling, water-gas shift, and electrosynthesis.^6−9^ Therefore, the scalable construction and application of SACs have emerged as key topics important both for the academic environment and for the industry.^10−12^

Deposition of stable single atoms on nitrogen-framed pores of carbon and carbon nitrides is perhaps the most successful strategy existing today to prepare SACs.^13−15^ The polymeric semiconductor graphitic carbon nitride (C_3_N_4_) is, in fact, an optimal scaffold due to its nitrogen-rich structure. The inherent nitrogen-lined pores can accommodate the metal single atoms in a stable manner^16^*via* “strong metal–support interaction” (SMSI),^17,18^ and this can enhance the photocatalytic performance. Carbon nitrides and Ni salts have been elegantly employed in photoredox catalysis.^19−22^ At the same time, poly(heptazine imides) possessing alkali, alkaline-earth abundant, and transition metal cations have been extensively studied in organic photoredox catalysis and H_2_ evolution.^23−29^ Despite these efforts, structure–property–performance relationship in single-atom photocatalysis is rarely elaborated. It is also unclear which properties make single-atom catalysts special.

Typically, metal deposition is performed post-synthetically by impregnation of prepared C_3_N_4_ polymers with a metal salt, followed by metal reduction and/or thermal treatment.^30^ However, this often leads to an undesirable decrease in the total surface area of the final composite as well as potential metal clustering. An alternative approach to homogeneously introduce metals into the C_3_N_4_ framework comprises the addition of a metal salt during the synthesis of carbon nitride. To avoid demixing, it has been shown that it is just crucial to select a suitable miscible and reactive metal precursor that would undergo co-condensation with the C_3_N_4_ precursors to ensure the homogeneity of the final composite.^30^

From a practical standpoint, SACs can exhibit very sharp product selectivities, just as their homogeneous analogues. This makes them particularly suitable to overcome low selectivity patterns in many chemical transformations.^31,32^ Among those, the removal of leftover pharmaceutical contaminants from water remains as one of the most challenging chemical transformations.^33,34^ By using light and a photocatalyst, it is potentially possible to photo-oxidize organic pollutants to CO_2_ and H_2_O. However, during degradation, stable product fragments that are more toxic than the initial substrate are frequently formed, limiting the industrial and societal exploitation of this technology.^33^ For example, in the TiO_2_/carbon dot-mediated photocatalytic degradation of Gemfibrozil (a drug used to cure abnormal blood lipid levels and found in wastewater), the contaminant easily degrades, but benzene derivatives are formed and released in the “purified” water, creating acute toxicity.^35,36^ Catalysts showing an increased selectivity toward CO_2_ have not been reported so far, although this would enable a form of carbon mining from wastewater.^37,38^

In this work, we demonstrate that photocatalyst design involving atomically dispersed metal species at mesoporous graphitic carbon nitride (mpg-C_3_N_4_) enables control and observation of ligand–metal charge transfer. In particular, using a series of spectroscopic techniques, we reveal that due to different coordination environments of metal single atoms on the surface of mpg-C_3_N_4_ or in the bulk of the material, ligand-to-metal charge transfer (LMCT) or ligand-to-metal-to-ligand charge transfer (LMLCT) is operative. The selected mode of charge transfer determines the activity of the single-atom catalyst and, for the specific degradation of Gemfibrozil, it drives the selectivity toward enhanced formation of CO_2_. The results correlate with the selected mode of charge transfer in the designed single-atom materials and open new avenues for the understanding and rational design of single-atom photocatalysts.

## Materials and Methods

### Catalyst Preparation

Sodium tricyanomethanide (98%) was purchased from IoLiTec. AgNO_3_ (≥99%), CuCl_2_·2H_2_O (≥99%), cyanamide (99%), Ludox HS40 (40 wt % suspension in water), and NH_4_HF_2_ (95%) were purchased from Sigma-Aldrich. The preparation of metal tricyanomethanides was performed according to the procedure detailed below.

#### Preparation of Silver(I) Tricyanomethanide

A solution of AgNO_3_ (1.70 g, 0.01 mol) in water (10 mL) was added in one portion to a stirred solution of sodium tricyanomethanide (1.13 g, 0.01 mol) in water (10 mL). The mixture was maintained under stirring in the dark for 3 h. A white solid was separated by centrifugation at 4000 rpm, washed with water (3 × 10 mL), and dried in vacuum (7 mbar, 50 °C). Yield: 1.92 g, 97%.

#### Preparation of Copper(II) Tricyanomethanide

A solution of CuCl_2_·2H_2_O (1.70 g, 0.01 mol) in water (10 mL) was added in one portion to a stirred solution of sodium tricyanomethanide (1.13 g, 0.01 mol) in water (10 mL). The mixture was maintained under stirring in the dark for 3 h. A brown solid was separated by centrifugation at 4000 rpm, washed with water (3 × 10 mL), and dried in vacuum (7 mbar, 50 °C). Yield: 0.95 g, 39%.

#### Synthesis of mpg-C_3_N_4_ Catalysts Modified with Ag and Cu Single Atoms

The synthesis of Ag@mpg-C_3_N_4_ and Cu@mpg-C_3_N_4_ was performed modifying an established procedure.^30^ Cyanamide (3.0 g), metal tricyanomethanide, and a 40% aqueous dispersion of 12 nm SiO_2_ particles (Ludox HS40) (7.5 g) were mixed and heated at 70 °C under stirring for 16 h until water was completely evaporated. The resulting mixture was heated at a rate of 2.2 °C min^–^^1^ over 4 h to reach a temperature of 550 °C. The temperature was kept constant for another 4 h. The resulting brown-yellow powder was briefly ground and treated with an NH_4_HF_2_ solution (12 g in 50 mL of water) for 24 h to remove the silica template. The suspension was centrifuged and the precipitate was washed three times with distilled water and once with ethanol. Finally, the product was dried at 60 °C under vacuum overnight. For Ag1@mpg-C_3_N_4_, Ag2@mpg-C_3_N_4_, Cu1@mpg-C_3_N_4_, and Cu2@mpg-C_3_N_4_, 30 mg (0.15 mmol) and 105 mg (0.53 mmol) of silver(I) tricyanomethanide and 37 mg (0.15 mmol) and 131 mg (0.53 mmol) of copper(II) tricyanomethanide were added, respectively. The mass values of products obtained for Ag1@mpg-C_3_N_4_, Ag2@mpg-C_3_N_4_, Cu1@mpg-C_3_N_4_ and Cu2@mpg-C_3_N_4_ were 1.64, 1.35, 1.78, and 1.61 g, respectively.

#### Reference (Transition Metal-Free) mpg-C_3_N_4_

The procedure of preparing mpg-C_3_N_4_ was similar with that of Ag@mpg-C_3_N_4_ and Cu@mpg-C_3_N_4_ except that no metal tricyanomethanide was added. The mass of the product obtained was 1.48 g.

### Catalyst Characterization

Scanning electron microscopy (SEM) and energy-dispersive X-ray (EDX) images were obtained on a JSM-7500F (JEOL) at an accelerating voltage of 3 kV. EDX investigations were conducted using a Link ISIS-300 system (Oxford Microanalysis Group) equipped with a Si(Li) detector and an energy resolution of 133 eV. Transmission electron microscopy (TEM) studies were performed using a double Cs-corrected JEOL JEM-ARM200F (S)TEM operated at 80 kV equipped with a cold field emission gun. Powder X-ray diffraction (XRD) was performed on a Bruker D8 Advance diffractometer equipped with a scintillation counter detector with Cu Kα radiation (λ = 0.15418 nm). Elemental analysis was accomplished by combustion analysis using a Vario Micro device. An inductively coupled plasma optical emission spectroscopy (ICP-OES) study was performed using an Optima 8000 ICP-OES spectrometer (PerkinElmer). Nitrogen adsorption–desorption measurements were performed after degassing the samples at 150 °C for 20 h using a Quantachrome Quadrasorb SI-MP porosimeter at 77 K. The specific surface areas were calculated by applying the Brunauer–Emmett–Teller (BET) model to adsorption isotherms for 0.05 < *p*/*p*_0_ < 0.3 using the QuadraWin 5.05 software package. The pore size distribution was obtained by applying the quenched solid density functional theory (QSDFT) model for N_2_ adsorbed on carbon with a cylindrical pore shape at 77 K. The optical absorbance spectra were measured on a Shimadzu UV 2600 spectrophotometer equipped with an integrating sphere. The photoluminescence (PL) spectra were recorded and the quantum yield (QY) was measured using an FP-8300 fluorescence spectrometer. The excitation wavelength was set to 365 nm. The time-resolved PL spectra were obtained on a fluorescence lifetime spectrometer (FluoTime 250, PicoQuant) equipped with a PDL 800-D picosecond pulsed diode laser drive. The decay curves were fitted using a nonlinear method with a multicomponent decay law given by the general formula *I* (*t*) = *a*_1_ exp(−*t*/τ_1_) + *a*_2_ exp(−*t*/τ_2_) + *a*_1_ exp(−*t*/τ_3_). The electron paramagnetic resonance (EPR) study was conducted on a Bruker EMXnano benchtop X-Band EPR spectrometer. The following settings were used: center field, 3200 G; sweep width, 3000 G; receiver gain, 40 dB; modulation amplitude, 1.000 G; number of scans, 1; microwave attenuation, 25 dB (0.3162 mW); room temperature. The Mott–Schottky measurements were carried out with the Arbin electrochemical testing station (Arbin Instrument) in a standard three-electrode quartz cell. The working electrode was prepared as follows: 2 mg of sample was suspended in 0.2 mL of deionized water containing 0.02 mL of 5 wt % Nafion D-520 dispersion, and the mixture was then dispersed by ultrasonication and spread onto an fluorine-doped tin oxide (FTO) glass. After being dried naturally, the FTO glass was heated at 120 °C for 1 h. The prepared thin film was employed as a working electrode, with a platinum plate as a counter electrode and Ag/AgCl as a reference electrode (3 M KCl). A 0.5 M Na_2_SO_4_ aqueous solution was used as an electrolyte (pH = 8.2). The measurement was carried out upon a frequency of 10 kHz in a potential range from −1.0 to 0.4 V *vs* Ag/AgCl. The measured potentials *vs* Ag/AgCl were converted to the reversible hydrogen electrode (RHE) scale according to the Nernst equation *E*_RHE_ = *E*_Ag/AgCl_ + *E*^0^_Ag/AgCl_ + 0.059 pH, where *E*_RHE_ is the converted potential *vs* RHE, *E*^0^_Ag/AgCl_ = 0.1976 at 25 °C, and *E*_Ag/AgCl_ is the experimentally measured potential against the Ag/AgCl reference. X-ray photoelectron spectroscopy (XPS) measurements were carried out with an X-ray gun Mg Kα radiation (1254.6 eV) using the CISSY end-station under ultra-high vacuum (UHV) at 1.5 × 10^–8^ Pa, equipped with a SPECS XR 50 and Combined Lens Analyzer Module (CLAM). The binding energy scale and Fermi level were calibrated using a gold film. The XPS quantitative analysis was performed through CasaXPS software using Lorentzian–Gaussian functions and Shirley background deletion in the photoemission spectra. The ultraviolet photoelectron spectroscopy (UPS) spectra were acquired with a He I (21.2 eV) radiation source. The detector was a combined lens with an analyzer module thermoVG (TLAM).

### Catalyst Testing

The photocatalytic performance of the prepared samples was studied using a solution of Gemfibrozil (10 mg) in deionized water (100 mL). This corresponds to a Gemfibrozil concentration of 100 ppm. All experiments were carried out using visible light (450 nm) irradiation. For the continuous-flow reactions, the water sample containing the pharmaceutical contaminant was pumped using a peristaltic pump through a custom-made photocatalytic reactor featuring a transparent column with a thin layer of catalyst (100 mg, with 0.2–0.3 mm particle size). The LEDs extended vertically along each side of the reactor and illuminated the central manifold where the packed catalyst layer was present. The experimental conditions (temperature, pressure, and residence time) were varied to explore their effect on the pollutant degradation. For the case of batch experiments, Gemfibrozil (10 mg) and the catalyst (100 mg, nanopowder) were placed in deionized water (100 mL) and the suspension was placed into a round-bottom flask and irradiated using LED lamps placed along each side of the flask. The product solutions were analyzed by a high-performance liquid chromatograph (Waters 1525 Binary HPLC pump). The stationary phase consisted of a Purospher Star RP-18 column (250 mm × 4.6 mm, 5 μm). The eluent phase was a mixture of water and methanol with a gradient concentration at a flow rate of 1.0 mL min^–1^. The concentration, conversion, and selectivity were calculated by a peak area method. To determine the concentration of organic moiety in water, product solutions were also measured by elemental CHN analysis (2400 CHN, PerkinElmer).

## Results and Discussion

### Composition and Textural Characterization of the Catalysts

A traditional polymerization method yields g-C_3_N_4_ with a low surface area (below 10 m^2^ g^–1^), resulting in limited activity in heterogeneous catalytic reactions.^39^ Therefore, in this work, a “hard” templating synthetic route using SiO_2_ nanoparticles has been chosen as an effective technique to obtain an ordered mesoporous structure. We have prepared, characterized, and evaluated a series of photocatalysts based on mpg-C_3_N_4_, containing isolated Ag and Cu single atoms on it. The introduction of silver and copper ions into a C_3_N_4_ network has been accomplished by co-condensation of the respective tricyanomethanide salts with cyanamide in the presence of 12 nm SiO_2_ nanoparticles as a hard template at 550 °C. During this process, the matrix nucleates and grows, embedding into the template. Removal of the template by treatment with (NH_4_)HF_2_ gives the mesoporous structure of the material with a high surface area, *i.e.*, the template replica. The resultant mesostructured Ag/Cu mpg-C_3_N_4_ with ordered porosity is expected to provide sufficient surface active sites and enhance light multireflection, to enable efficient photocatalytic reactions. The final composition and textural properties of the reference and single-atom mesoporous graphitic carbon nitrides are shown in Table 1. Assuming the ideal C_3_N_4_ molecular formula for the synthesized organic part of the material, the metal content in Ag@mpg-C_3_N_4_, *i.e.*, 0.31–0.32 wt %, suggests that statistically one silver atom is surrounded by *ca.* 100 heptazine units. In the case of Cu@mpg-C_3_N_4_, the concentration of metal single atoms is higher, and for Cu1@mpg-C_3_N_4_, it is one atom per 70 heptazine units, while for Cu2@mpg-C_3_N_4_, it is one atom per 20 units. The percentage of metal transferred from the tricyanomethanide precursor into the material is higher for Cu (>79%) compared to Ag (7–32%). In particular, it is quantitative for Cu1@mpg-C_3_N_4_ (Table S2). Independent from the presence of isolated metal species on it, the mesoporous structure of the prepared materials is confirmed (Table 1).

**Table 1:** Elemental Composition and Textural Properties of the Reference Mesoporous Graphitic Carbon Nitride and Single Atom-Based Catalysts

catalyst	C^a^ [wt %]	N^a^ [wt %]	H^a^ [wt %]	C/N [−]	Ag^b^ [wt %]	Cu^b^ [wt %]	*S*_BET_^c^ [m^2^ g^–1^]	*V*_pore_^d^ [cm^3^ g^–1^]
mpg-C_3_N_4_	31.90 ± 0.12	48.75 ± 0.39	2.43 ± 0.04	0.65			157	0.46
Ag1@mpg-C_3_N_4_	30.08 ± 0.46	47.31 ± 0.08	2.61 ± 0.02	0.64	0.32 ± 0.02		127	0.25
Ag2@mpg-C_3_N_4_	31.37 ± 0.01	47.37 ± 0.08	2.30 ± 0.02	0.66	0.31 ± 0.02		232	0.60
Cu1@mpg-C_3_N_4_	31.58 ± 0.02	49.05 ± 0.04	2.47 ± 0.02	0.64		0.54 ± 0.01	274	0.72
Cu2@mpg-C_3_N_4_	32.27 ± 0.18	47.96 ± 0.18	2.33 ± 0.04	0.67		1.66 ± 0.03	257	0.71

a C, N, and H elemental analysis. The complete analysis is given in Table S1 in the Supporting Information.

b ICP-OES data.

c BET method applied to the N_2_ isotherm collected at 77 K.

d Quenched solid density functional theory model assuming cylindrical-shaped pores for the reference mpg-C_3_N_4_ and single-atom catalysts.

**Figure 1:**
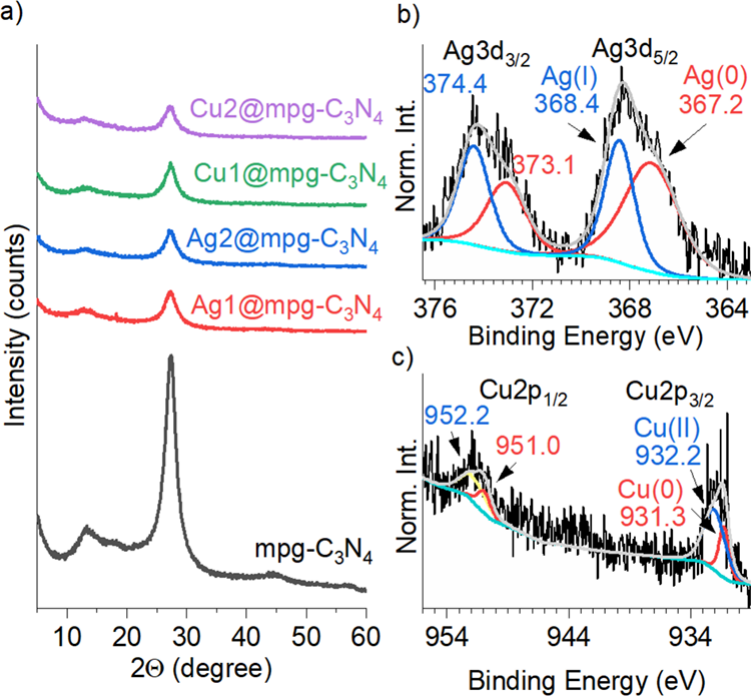
(a) X-ray diffraction patterns of the different catalysts. (b) Ag 3d XPS of Ag2@mpg-C_3_N_4_. (c) Cu 2p XPS of Cu2@mpg-C_3_N_4_.

The chemical structure of the final C_3_N_4_-based catalysts is similar to that of the reference mesoporous graphitic carbon nitride, as revealed by the X-ray diffraction patterns (Figure 1a). All mpg-C_3_N_4_ samples have two characteristic diffraction peaks, at 13° and 27°. The former is related to an in-plane structural packing motif; the latter is attributed to the interplanar stacking of aromatic systems identified as the (002) peak.^40,41^ Single metal atoms disturb the local structure of the C_3_N_4_ network as illustrated by an intensity decrease and slight broadening of the stacking diffraction peak at 27° 2θ, suggesting an increased distortion of the stacking arrangement of the carbon nitride layers upon an increase in the number of Ag and Cu precursors.

### Nature of the Induced Chemical Changes in the C_3_N_4_ Network

X-ray photoelectron spectroscopy (XPS) was performed followed by fitting with Lorentzian–Gaussian curves and analysis (Figures S2 and S3). Quantitative analysis from the XPS spectra reveals that the C/N atomic ratio of the samples is *ca*. 0.66 (Table S3), in line with the total C/N ratio. In the XPS C 1s spectra, a peak at 284.0 eV (in the literature, mainly calibrated at 284.8 eV and defined as “adventitious carbon”)^42^ is assigned to electron-rich, *e.g.*, carbide-like, carbon (C_carb_).^30^ The peak at 287.4 eV is assigned to carbon bound to terminal NH_2_ groups (C–NH_2_). The peak at 288.7 eV is assigned to N—C=N moieties in heptazine rings.^41^ The N 1s peak can be deconvoluted into three peaks centered at 398.8, 399.5, and 401.0 eV, corresponding to pyridinic N (C=N—C), tertiary nitrogen (N–(C)_3_), and amino functional groups (C–NH_2_ and C–NH–C), respectively.^43^ In general, introduction of either Ag or Cu single atoms leads to the shift of the C_carb_ peak by 0.5–0.8 eV to higher energies,^44^ indicating that metal single atoms bind selectively to electron-rich sites in the carbon nitride and induce redistribution of electron density in the material.

XPS of Ag 3d exhibits peaks at 368.4 and 367.2 eV, which are characteristic of Ag(I) and Ag(0), respectively (Figure 1b). Similarly, from XPS, Cu 2p peaks at 932.2 and 931.3 eV assigned to Cu(II) and Cu(0), respectively, can be indexed (Figure 1c). The presence of Ag(0) and Cu(0) peaks along with Ag(I) and Cu(II) complements XPS C 1s data and indicates partial charge transfer between the carbon nitride framework and the metal species. XPS, in fact, is a technique that is able to establish the occurrence of charge transfer mechanisms in catalysts, even in the presence of electron acceptor and donor ligands, molecules, and dopants.^45^ In particular, to quantitatively evaluate charge transfer effects and estimate the extent, it is possible to calculate the ratio of the areas under the XPS peaks, which corresponds to charged species (*i.e*., which have donated/accepted electrons) to that for C 1s, correcting for different cross sections for radiation. Elsewhere, it has been reported that Cu single atoms deposited at carbon nitride^46,47^ and graphene^48,49^ carry partial positive charge. Based on integrated areas of the peaks, in Ag2@mpg-C_3_N_4_, we estimate that 0.61*e* is transferred to silver (Figure 1b), while in Cu2@mpg-C_3_N_4_, 0.86*e* is transferred to copper (Figure 1c), which quantify for the first time the extent of charge transfer in single-atom catalysts.

**Figure 2:**
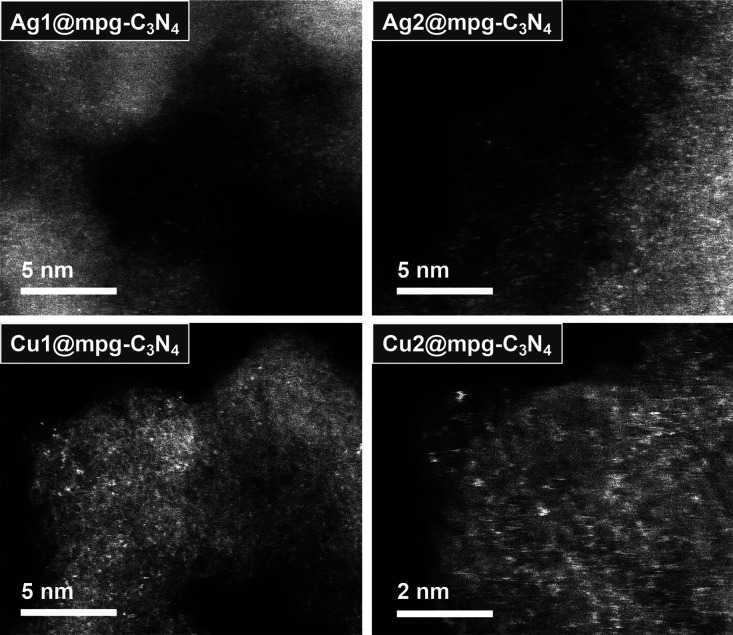
Aberration-corrected high-resolution TEM images of the catalysts show isolated single atoms distributed across the mpg-C_3_N_4_ support.

The morphology of the single-atom catalysts is similar to the reference mpg-C_3_N_4_ and is represented by particles with a diameter of *ca.* 20–200 nm as seen from the SEM images (Figure S4). TEM images show the presence of abundant mesopores, which originate from the removal of the template (Figure S5). These have an average pore size of *ca.* 5–15 nm, which is in line with the results of N_2_ physisorption studies, showing type IV isotherms and type H3 hysteresis loops (Figure S1). The energy-dispersive X-ray spectra of all materials retrieved from the collected SEM and TEM maps confirm the presence of all relevant chemical elements (Figures S6–S11). High-angle annular dark-field scanning transmission electron microscopy (HAADF-STEM) investigation performed on an aberration-corrected microscope showed the presence of silver and copper elements in an atomically dispersed form throughout the samples (Figure 2). The absence of any regions of higher local intensity can be confirmed in the Ag and Cu micrographs, verifying the absence of aggregated clusters and crystalline metal nanoparticles. Overall, the results of TEM study and XPS data point to the presence of an ensemble of Ag and Cu atoms in a fractional oxidation state, which makes the prepared materials a joint electronic system modified with metal single atoms rather than isolated metal single sites only dropped onto a support. This confirms that the strong interaction between the support and metal sites is the key to the fabrication of single-atom catalysts and it is impossible to produce single-atom catalysts by simply “dropping” the metals on the cavities of the support.

**Figure 3:**
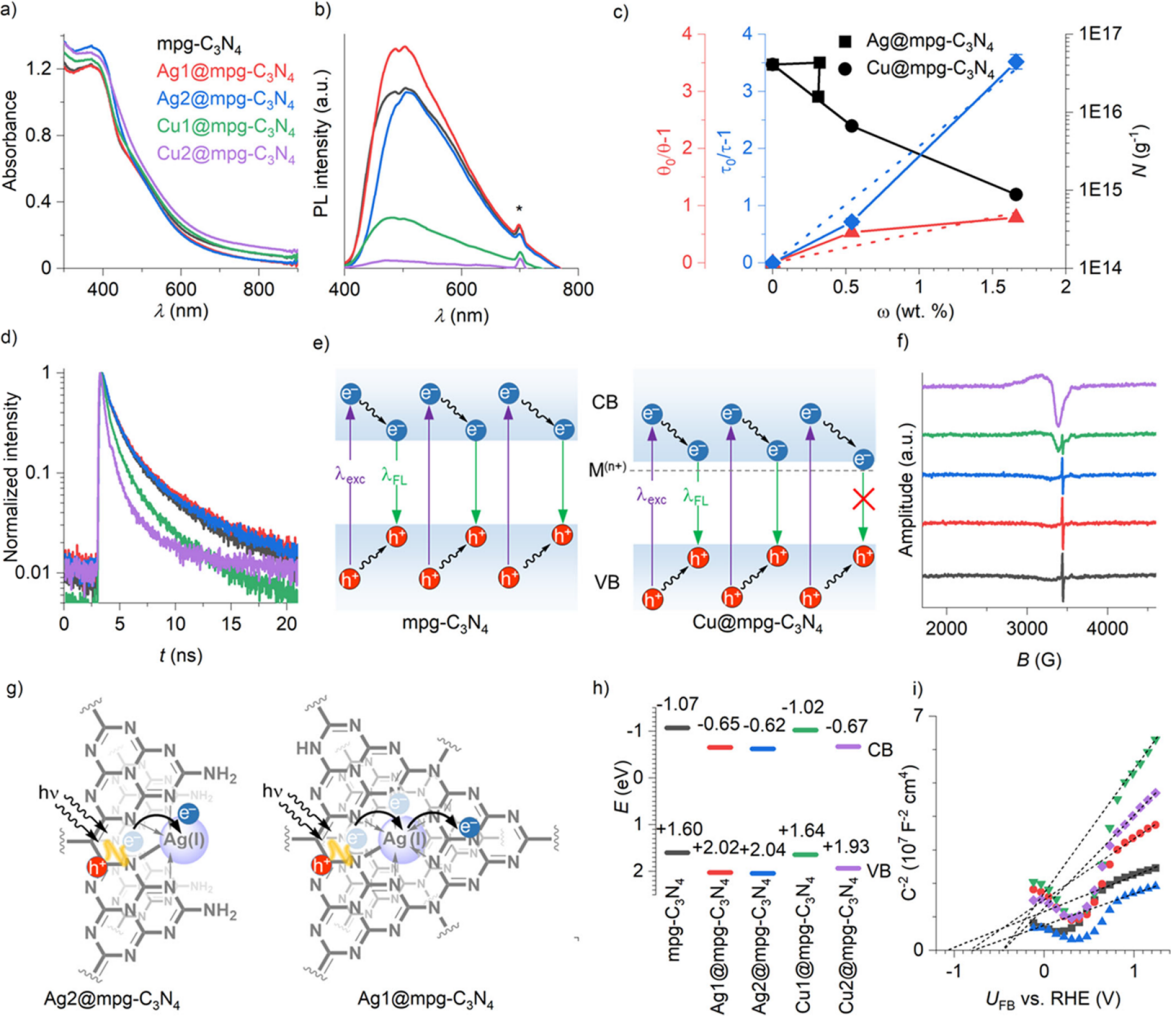
Advanced characterization of the materials. (a) DRUV–vis absorption spectra. The color scheme is the same for the panels (a), (b), (d), (f), (h), and (i) shown in this figure. (b) Steady-state emission spectra of the materials recorded upon sample excitation with λ = 350 nm. The asterisk denotes second-order excitation light diffraction. (c) The structure–property relationship shows the dependence of physico-chemical properties of materials versus metal single-atom content. (d) TRES upon sample excitation with λ = 375 nm. (e) Schematic representation of the effect of metal single atoms on the band structure of the materials and excited-state dynamics. The reference mpg-C_3_N_4_ and Cu@mpg-C_3_N_4_ are exemplified. (f) Room-temperature X-band EPR spectra of materials in the solid state. (g) Schematic representation of LMCT in Ag2@mpg-C_3_N_4_ and LLCT in Ag1@mpg-C_3_N_4_. (h) Band structure of the materials based on UPS and DRUV–vis absorption spectroscopy. (i) Mott–Schottky plots.

The influence of metal single atoms on the electronic properties of the C_3_N_4_ structure was first examined by diffuse reflectance UV–vis (DRUV–vis) absorption spectroscopy (Figure 3a). All samples absorb in the visible region of the electromagnetic spectrum. In particular, the reference mpg-C_3_N_4_ shows an absorption edge at *ca.* 460 nm (equivalent to the absorption of photons with energy > 2.7 eV), which originates from π–π* transitions (Figure 3a).^40,41^ Introduction of metal single atoms into the mpg-C_3_N_4_ has a dual effect on light absorption properties of the material. First, the optical gap narrows by *ca.* 0.01–0.07 eV (Figure S12). This effect is the most pronounced for Cu2@mpg-C_3_N_4_ and is apparently due to the highest Cu content, 1.66 wt %, in the studied series of materials. Second, light absorption in the range of 500–800 nm is enhanced due to the introduction of metal ions, which facilitate LMCT. Charge transfer between carbon nitride and metal atoms is further confirmed by quenching of mpg-C_3_N_4_ fluorescence (Figure 3b). The fluorescence internal quantum efficiency (IQE, θ) decreases from 0.65% for pristine mpg-C_3_N_4_ to 0.59% for Ag2@mpg-C_3_N_4_ and 0.36% for Cu2@mpg-C_3_N_4_ (Table S4). For comparative studies, we have selected materials modified with Cu because of similar textural properties, *i.e.*, surface area and specific pore volume (Table 1), which eliminates the influence of these parameters on photophysical properties.^50,51^ The influence of Cu single atoms on fluorescence quenching was quantitatively evaluated using the Stern–Volmer approach (Figure 3c). Thus, for Cu@mpg-C_3_N_4_, the Stern–Volmer constant *K*_SV_ was calculated to be 0.53 (wt %)^−1^.^52^ The results of steady-state fluorescence quenching are supported by the time-resolved emission spectra (TRES) (Figure 3d and Table S5). Thus, photogenerated charge carriers in Cu2@mpg-C_3_N_4_ have a *ca.* 5 times shorter lifetime compared to the reference mpg-C_3_N_4_, namely, 172 ± 3 ps versus 778 ± 18 ps. The fluorescence quenching rate constant *k*_q_ was calculated to be (2.63 ± 0.06) × 10^9^ (wt %)^−1^ s^–1^. Despite the fact that excitons in the reference mpg-C_3_N_4_ possess already very short lifetime compared, for example, to solutions of molecular photocatalysts,^53^ introduction of Ag and Cu is still able to efficiently quench fluorescence, because single atoms are within the subnanometer distance from the fluorophore, which facilitates their interaction, while diffusion as a potential rate-limiting step is eliminated.^54^ Taking into account low IQE for the set of the synthesized materials and TRES data, we conclude that excitons in mpg-C_3_N_4_ reach surface states in less than 0.8 ns, while those in Cu2@mpg-C_3_N_4_ reach surface states in less than 0.2 ns. In the notation adopted in the semiconductor community, metal single atoms are considered as unoccupied defect states located close to the conduction band (CB) edge (Figure 3e).^55^ In the studied materials, these surface states are nonfluorescent as seen from the steady-state emission spectra—no additional peaks are observed, while emission maxima are observed at *ca.* 500 nm (Figure 3b).

Electron paramagnetic resonance (EPR) spectroscopy was employed to probe the unpaired electrons in the catalysts (Figure 3f). The reference mpg-C_3_N_4_ shows a sharp signal at 3440 G having line width of *ca*. 5 G associated with the presence of radical species in the π-conjugated structure of the material. Introduction of metal single atoms leads to gradual quenching of the signal as the content of metal increases. Quantitative EPR reveals an exponential decrease in the concentration of the radical species associated with the carbon nitride backbone (Figure 3c and Table S6). Materials with nearly identical textural properties, Ag2@mpg-C_3_N_4_, Cu1@mpg-C_3_N_4_, and Cu2@mpg-C_3_N_4_ (Table 1), show a monotonous decrease in the specific concentration of radical species regardless of the incorporated metal ion (Figure 3c). At the same time, Ag1@mpg-C_3_N_4_ having twice lower surface area and specific pore volume, but nearly the same content of Ag as Ag2@mpg-C_3_N_4_, falls out of this trend, implying that LMCT effectively proceeds only in case metal single atoms are localized on the surface of the material.^56^ For the same reason, Ag1@mpg-C_3_N_4_ shows even higher photoluminescence intensity (Figure 3b) and longer fluorescence lifetime compared to the reference mpg-C_3_N_4_ (Table S5). For Cu@mpg-C_3_N_4_ along with the quenching of the narrow signal, a broad peak covering the full spectral window evolves (Figure 3f). This signal is associated with Cu(II) paramagnetic centers, in agreement with the XPS data.^57^

Overall, spectroscopic data derived from steady-state emission spectroscopy, TRES, and EPR spectroscopy strongly point to LMCT. This process seems to occur only in the case of metal single atoms located on the surface of the material (Figure 3g). The possible reason consists of different coordination environments of surface metal single atoms and those located in the bulk of carbon nitride. Surface metal single atoms are coordinated by the carbon nitride from one side (a type of pincer complex), while the bulk metal single atoms are entrapped into a cage created by heptazine units (cryptand-like complex). Therefore, for surface metal single atoms, only unidirectional electron transfer, from carbon nitride to the metal site, is possible. On the contrary, bulk metal single atoms “conduct” electrons via LMLCT.^58,59^ Such a charge transfer process in Ag1@mpg-C_3_N_4_ occurs on the subpicosecond scale,^60^ while enhanced electron mobility is registered as the amplified EPR signal (Table S6), enhanced fluorescence (Figure 3b), and extended lifetime of the excitons (Figure 3d).

To investigate the influence of metal single atoms on the alignment of the energy levels, we determined the valence band (VB) energies using ultraviolet photoelectron spectroscopy (UPS) (Figure S13). Introduction of metal single atoms results in the shift of the VB by 0.04–0.45 eV to more positive numbers compared to pristine mpg-C_3_N_4_ (Figure 3h). The influence is more pronounced for Ag@mpg-C_3_N_4_ compared to Cu@mpg-C_3_N_4_. Subtraction of optical gap values derived from the Kubelka–Munk function from the VB energies gave CB energies of the materials, which are lower in energy compared to the reference mpg-C_3_N_4_ (Figure 3h). We also applied the Mott–Schottky analysis to study the influence of metal single atoms onto the quasi-Fermi level of electrons in the semiconductors (Figure 3i). All samples show a positive slope, which is characteristic for *n*-type semiconductors with electrons as major carriers. The flat band potential (*U*_FB_) of mpg-C_3_N_4_ was determined to be −1.05 V *vs* RHE, while for mpg-C_3_N_4_ modified with Ag and Cu single atoms, the corresponding *U*_FB_ values are shifted to more positive values, *i.e.*, *ca.* −0.81 V and −0.46 V *vs* RHE, respectively (Figure 3i). Overall, our data indicate that LMCT and LMLCT arise from transfer of charges from the metal to the carbon nitride, which acts as a ligand. This type of transfer is predominant in homogeneous complexes with relatively high energy lone pairs (*e.g.*, O, S, or Se) or when the metal has low empty orbitals but, to our knowledge, was never experimentally observed over single-atom catalysts.

### Charge Transfer Effects on the Photocatalytic Degradation of Gemfibrozil

**Figure 4:**
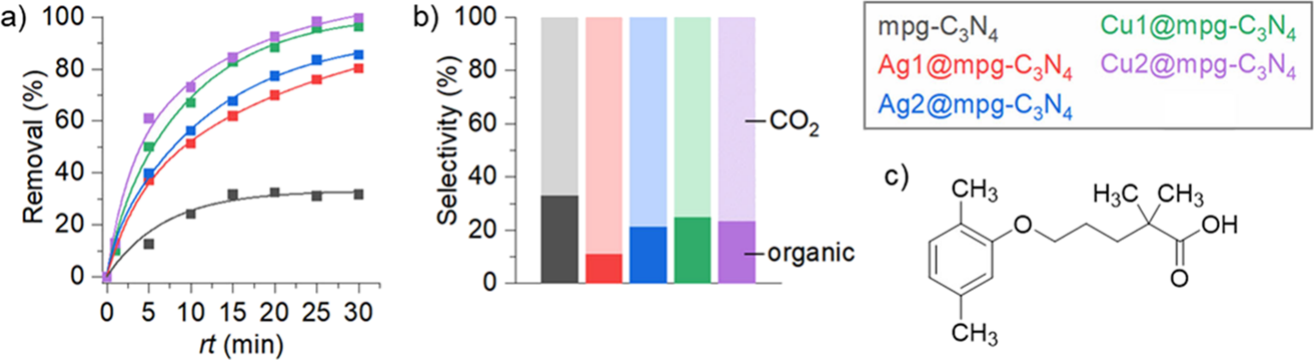
(a) Influence of the photoreactor residence time (*rt*) on the continuous-flow degradation of Gemfibrozil in water over pristine mpg-C_3_N_4_ and metal-based single-atom catalysts. Conditions: [Gemfibrozil] = 100 ppm, *m*_cat_ = 100 mg, temperature = 30 °C, pressure = 3 bar, and light wavelength = 450 nm. (b) Selectivity to CO_2_ and number of toxic organic byproducts over pristine mpg-C_3_N_4_ and metal-based single-atom catalysts. Conditions: [Gemfibrozil] = 100 ppm, *m*_cat_ = 100 mg, temperature = 30 °C, pressure = 3 bar, *rt* = 1–5 min (at a reference Gemfibrozil removal = 10%), and light wavelength = 450 nm. (c) Structure of Gemfibrozil.

Using the materials described above, we have studied the photocatalytic removal of Gemfibrozil from deionized water. Gemfibrozil is a major contaminant present in water samples and is among the emerging pollutants listed on the 3rd Watch List under the Water Framework Directive of the European Union.^61^ This compound is hard to degrade using state-of-the-art technologies^62^ and, when this happens, toxic organic moieties that are stable and soluble in water are formed.^35,36^ Our catalytic tests have been conducted under continuous-flow conditions. As shown in Figure 4a, the removal of Gemfibrozil increases with longer residence time (*rt*) in the photoreactor until it reaches a plateau when *rt* > 25 min. Such a trend is common and has been reported elsewhere.^63,64^ In fact, a heterogeneous photocatalytic reaction involves adsorption of reactants from a fluid phase onto a solid surface, surface reaction of the adsorbed species, and desorption of products into the fluid phase. Clearly, substances other than the reactants, including impurities, reaction intermediates, or (by)products, can also be adsorbed on the catalyst surface and inhibit the reaction due to partial occupation of the catalyst active sites, resulting in a decrease in the degradation rate. It is important to remark, however, that this effect is more pronounced over pristine mpg-C_3_N_4_ and less over the single-atom catalysts.

The order of reactivity is Cu2@mpg-C_3_N_4_ > Cu1@mpg-C_3_N_4_ > Ag2@mpg-C_3_N_4_ > Ag1@mpg-C_3_N_4_ > mpg-C_3_N_4_. This effect does not change after fixing the residence time and varying the temperature or the pressure (Figure S14) or using a slurry batch-type photoreactor (Figure S15). Also, the degradation takes place only in the presence of light. Finally, a stable photocatalytic performance is observed for all materials (Figure S16).

Based on the data in Figure 4a, it is possible to model the photodegradation of Gemfibrozil in water using the equation *c* = *c*_0_ exp(−*kt*), where *c* represents the concentration of Gemfibrozil over time, and *c*_0_ is its initial concentration in water. This equation represents the best practice to simulate the photoreactor behavior, considering that an in-depth analysis of Gemfibrozil degradation through *ab initio* simulations is not yet available. In particular, the equation corresponds to a pseudo-first-order concentration decay, which is well applicable since the curves of ln(*c*/*c*_0_) versus retention time (*rt*) show a nearly linear relationship (Figure S17). From this equation, the rate constant (*k*) and estimated pollutant half-life (*t*_1/2_) have been determined (Table S7) to provide valuable kinetic insights. By comparing the rate constants with values available in the literature (Table S8), it is possible to appreciate the efficiency of the single-atom materials. The estimated half-life for the contaminant degradation is in line with this result and further demonstrates that Cu2@mpg-C_3_N_4_ requires only 4 min to halve the contaminant concentration.

The photocatalytic performance can be correlated with the above-discussed photophysical properties of the materials influenced by the introduction of single atoms (Figure 3c). Thus, fluorescence lifetime in combination with relatively low IQE (<1% for all materials studied herein) are seen as the parameters characterizing the efficiency of charge separation and localization at nonradiative surface states. This process is the fastest for Cu2@mpg-C_3_N_4_ (τ = 172 ps), which correlates with the highest activity in the degradation of Gemfibrozil. The nature of metal single atoms has a clear influence on the performance of the photocatalyst. Upon photoexcitation of Cu@mpg-C_3_N_4_, LMCT promotes the reduction of Cu(II) to Cu(I)/Cu(0) mononuclear complexes. The latter activates oxygen via SET and therefore accelerates the degradation of the pollutant.^65−67^ A similar process in Ag@mpg-C_3_N_4_ leads to the more stable Ag(0) species (*E*_Ag(I)/Ag(0)_ = +0.8 V *vs* SHE). Therefore, metal single atoms not only adjust the photophysical properties of the carbon nitride semiconductor but also are efficiently involved in the process of Gemfibrozil degradation.

To unravel the product selectivity formed over the different photocatalysts, we have determined the organic content in the product solutions via CHN analysis (Figure 4b). Organic moieties, owing to the incomplete conversion of the Gemfibrozil core, are formed during the degradation. However, from the carbon balance, we estimate that a higher fraction of gaseous species and fewer organic byproducts are formed in the case of using Ag@mpg-C_3_N_4_ and Cu@mpg-C_3_N_4_ compared to the reference mpg-C_3_N_4_. Ag1@mpg-C_3_N_4_ clearly stands out as the most selective catalyst toward CO_2_, followed by Ag2@mpg-C_3_N_4_. Despite the fact that Cu2@mpg-C_3_N_4_ showed the highest activity in the removal of Gemfibrozil, its selectivity toward CO_2_ is moderate, suggesting the existence of a follow-up cascade, *i.e.*, the drug molecule undergoes transformation quickly, but calcination is a slower process. Note that the catalysts were compared at a similar degree of conversion and under kinetic conditions (10%). These results point that single-atom photocatalysts can reduce the amount of toxic species of benzene during the pollutant degradation. Although Ag1@mpg-C_3_N_4_ and Ag2@mpg-C_3_N_4_ have nearly the same Ag content, their textural properties are different (Table 1). As deduced from the spectroscopic study, bulk Ag single atoms in Ag1@mpg-C_3_N_4_ facilitate electron transport that is beneficial to promote the multistep pathway of Gemfibrozil degradation to CO_2_.

## Conclusions

We have prepared, characterized, and evaluated a series of single-atom heterogeneous catalysts featuring highly dispersed metal species on mesoporous graphitic C_3_N_4_. Using this series of catalysts, we have shown that Ag and Cu single metal species can decrease the extent of aromatic byproducts in the degradation of emerging water contaminants and promote the selective formation of CO_2_. By combining advanced characterization methods, we show that such results can be linked to ligand–metal charge transfer in the designed materials, as confirmed by DRUV–vis, TRES, fluorescence quantum efficiency, and EPR measurements. Specifically, textural properties of mpg-C_3_N_4_ modified with metal single atoms influence the mode and type of charge transfer in the studied materials. Overall, this work not only shows the incorporation and use of Cu and Ag atoms on carbon nitride for photocatalytic water decontamination but also opens fundamental directions in the design of selective single-atom photocatalysts, showing how fine-tuning the band structure of the hybrid semiconductor nanomaterials can lead to improved photocatalytic data.

## Supporting Information Available

Materials elemental characterization of the reference mesoporous graphitic carbon nitride and single-atom catalysts based on CHNS analysis; percentage of metal transferred from tricyanomethanides into the carbon nitride materials; materials elemental characterization of the reference mesoporous graphitic carbon nitride and single-atom catalysts based on XPS analysis; quantum yield for the reference mesoporous graphitic carbon nitride and single-atom catalysts; fluorescence lifetime measured over the reference mesoporous graphitic carbon nitride and single-atom catalysts; quantitative EPR data; kinetic parameters obtained for the photocatalytic degradation of Gemfibrozil over the reference mesoporous graphitic carbon nitride and single-atom catalysts; literature precedents for the degradation of emerging pharmaceutical contaminants using similar carbon nitride materials; nitrogen sorption isotherms at 77K of samples and corresponding pore size distribution calculated by a QSDFT model assuming cylindrical-shaped pores of samples; C 1s, N 1s, and Ag 3d X-ray photoelectron spectroscopy of mpg-C_3_N_4_, Ag1@mpg-C_3_N_4_, and Ag2@mpg-C_3_N_4_; C 1s, N 1s, and Cu 2p X-ray photoelectron spectroscopy of mpg-C_3_N_4_, Cu1@mpg-C_3_N_4_, and Cu2@mpg-C_3_N_4_; scanning electron microscopy of the catalysts; high-resolution transmission electron microscopy of the single-atom catalysts; TEM-EDX spectrum of Cu2@mpg-C_3_N_4_; SEM-EDX elemental analyses of mpg-C_3_N_4_, Ag1@mpg-C_3_N_4_, Ag2@mpg-C_3_N_4_, Cu1@mpg-C_3_N_4_, and Cu2@mpg-C_3_N_4_; Tauc plots for the materials; UPS spectra of the materials and extended UPS spectra of the materials; effect of temperature and pressure on the degradation of Gemfibrozil in water over mpg-C_3_N_4_, Ag1@mpg-C_3_N_4_, Cu1@mpg-C_3_N_4_, and Cu2@mpg-C_3_N_4_; removal of Gemfibrozil in water over different photocatalysts under batch-type conditions; catalyst performance as a function of the time-on-stream; pseudo-first-order kinetic fitting curves (PDF)
