# Supplementary material for: Ligand–Metal Charge Transfer Induced via Adjustment of Textural Properties Controls the Performance of Single-Atom Catalysts during Photocatalytic Degradation
Source: ACS Appl Mater Interfaces. 2021 May 24;13(22):25858–67. doi: 10.1021/acsami.1c02243 (PMC8289176; doi:10.1021/acsami.1c02243)
Supplement: Supplementary file 1 — am1c02243_si_001.pdf [file am1c02243_si_001.pdf]

Supporting Information

Ligand-Metal Charge Transfer Induced *via* Adjustment of Textural Properties Controls the Performance of Single-Atom Catalysts During Photocatalytic Degradation

Jiaxu Liu,‡[a,b] Yajun Zou,‡[c] Daniel Cruz,[d,e] Aleksandr Savateev,[c] Markus Antonietti,[c] and Gianvito Vilé*[a]*

[a] Department of Chemistry, Materials, and Chemical Engineering "Giulio Natta", Politecnico di Milano, Piazza Leonardo da Vinci 32, 20133 Milan (Italy).

[b] State Key Laboratory of Fine Chemicals, Department of Catalytic Chemistry and Engineering, Dalian University of Technology, Ganjingzi District, Linggong Road 2, 116024 Dalian (China).

[c] Department of Colloid Chemistry, Max-Planck Institute of Colloids and Interfaces, Potsdam-Golm Science Park, Am Mühlenberg 1 OT Golm, 14476 Postdam (Germany).

[d] Department of Inorganic Chemistry, Fritz-Haber-Institut der Max-Planck-Gesellschaft, Faradayweg 4-6, 14195 Berlin (Germany).

[e] Department of Heterogeneous Reactions, Max Planck Institute for Chemical Energy Conversion, Mülheim an der Ruhr (Germany).

‡ These two authors contributed equally.

*Corresponding authors. E-mails: gianvito.vile@polimi.it (G.V.)
oleksandr.savatieiev@mpikg.mpg.de (A.S.)

Table S1. Materials elemental characterization of the reference mesoporous graphitic carbon nitride and single-atom catalysts based on CHNS analysis. For each sample, two different measurements were taken. The average data are indicated in ***italics***.

Catalyst	C (wt.%)	N (wt.%)	H (wt.%)	S (wt.%)	C/N (-)	C/H (-)
mpg-C_3N_4	32.02	49.14	2.46	0.26	0.65	13.03
	31.78	48.36	2.39	0.13	0.66	13.28
	31.90	***48.75***	***2.43***	***0.20***	***0.66***	***13.66***
Ag1@mpg-C_3N_4	30.54	47.39	2.58	0.10	0.64	11.85
	29.62	47.22	2.63	0.09	0.63	11.24
	30.08	***47.31***	***2.61***	***0.10***	***0.64***	***11.55***
Ag2@mpg-C_3N_4	31.36	47.45	2.28	0.08	0.66	13.76
	31.37	47.28	2.32	0.06	0.66	13.54
	31.37	***47.37***	***2.30***	***0.07***	***0.66***	***13.65***
Cu1@mpg-C_3N_4	31.56	49.09	2.45	0.05	0.64	12.87
	31.60	49.00	2.48	0.05	0.64	12.73
	31.58	***49.05***	***2.47***	***0.05***	***0.64***	***12.80***
Cu2@mpg-C_3N_4	32.09	47.78	2.29	0.04	0.67	14.02
	32.44	48.14	2.36	0.05	0.67	13.76
	32.27	***47.96***	***2.33***	***0.05***	***0.67***	***13.89***

Table S2. Percentage of metal transferred from tricyanomethanides into the carbon nitride materials.

Catalyst	Mass of metal in the mixture of precursors, m_p (g)	Mass of the metal in the single-atom catalyst, m_m[a] (g)	Efficiency of metal transferring from the precursor to the catalyst[b] (%)
Ag1@mpg-C_3N_4	0.016	0.0052	32
Ag2@mpg-C_3N_4	0.057	0.0042	7
Cu1@mpg-C_3N_4	0.0096	0.0096	100
Cu2@mpg-C_3N_4	0.034	0.0270	79

[a] Based on ICP-OES.

[b] Calculated as ratio $\frac{m_m}{m_p}$ ×100%.

Table S3. Materials elemental characterization of the reference mesoporous graphitic carbon nitride and single-atom catalysts based on XPS analysis.

Catalyst	C (wt.%)	N (wt.%)	C/N (-)	Ag (wt.%)	Cu (wt.%)
mpg-C_3N_4	39.95±0.45	60.05±0.45	0.665	-	-
Ag1@mpg-C_3N_4	38.99±0.53	60.71±0.53	0.642	0.30±0.09	-
Ag2@mpg-C_3N_4	40.59±0.33	59.08±0.33	0.687	0.33±0.03	-
Cu1@mpg-C_3N_4	40.23±0.56	59.77±0.57	0.673	-	-
Cu2@mpg-C_3N_4	39.69±0.50	59.81±0.50	0.664	-	0.50±0.21

Table S4. Quantum yield for the reference mesoporous graphitic carbon nitride and single-atom catalysts.

Catalyst	External quantum efficiency (%)	Internal quantum efficiency (%)
mpg-C_3N_4	0.584	0.651
Ag1@mpg-C_3N_4	0.583	0.648
Ag2@mpg-C_3N_4	0.531	0.587
Cu1@mpg-C_3N_4	0.383	0.424
Cu2@mpg-C_3N_4	0.326	0.363

Table S5. Fluorescence lifetime measured over the reference mesoporous graphitic carbon nitride and single-atom catalysts.[a]

Catalyst	τ_1 (a_1), ps (%)	τ_2 (a_2), ps (%)	τ_3 (a_3), ps (%)	τ,[b] ps	$\bar{\tau}$, ps
mpg-C_3N_4	4802 (7.1)	1111 (31.8)	171 (61.1)	798	778±18
	4893 (6.4)	1100 (31.3)	156 (62.3)	755	
	4838 (6.8)	1090 (32.3)	166 (60.9)	782	
Ag1@mpg-C_3N_4	4863 (8.2)	1126 (30.5)	158 (61.3)	839	843±5
	4860 (8.2)	1132 (30.3)	161 (61.5)	840	
	4957 (7.8)	1148 (31.3)	166 (60.8)	850	
Ag2@mpg-C_3N_4	4884 (6.8)	1095 (31.3)	152 (61.9)	770	758±9
	4840 (6.7)	1113 (29.7)	154 (63.6)	754	
	4802 (6.8)	1120 (28.6)	156 (64.6)	749	
Cu1@mpg-C_3N_4	4200 (3.5)	883 (21.8)	139 (74.7)	443	453±9
	4140 (3.7)	898 (21.5)	142 (74.8)	453	
	4160 (3.7)	901 (22.6)	142 (73.7)	737	
Cu2@mpg-C_3N_4	4590 (0.6)	831 (8.4)	79 (91.0)	170	172±3
	3740 (0.9)	824 (8.1)	77 (91.0)	171	
	3970 (0.9)	798 (8.7)	80 (90.4)	176	

[a] The data was acquired with excitation wavelength of 375 nm and emission wavelength of 530 nm.

[b] Calculated as $\frac{\sum a_i \tau_i}{\sum a_i}$.

Table S6. Quantitative EPR data.[a]

Catalyst	N (g^{-1})
mpg-C_3N_4	410×10^{14}
Ag1@mpg-C_3N_4	434×10^{14}
Ag2@mpg-C_3N_4	159×10^{14}
Cu1@mpg-C_3N_4	67×10^{14}
Cu2@mpg-C_3N_4	9×10^{14}

[a] Calculated for a signal at 3440 G.

Table S7. Kinetic parameters obtained for the photocatalytic degradation of Gemfibrozil over the reference mesoporous graphitic carbon nitride and single-atom catalysts. The analysis refers to the results in Figure 4a in the main manuscript.

Catalyst	Kinetic equation	k (min^{-1})	$t_{1/2}$ (min)	Correlation coefficient R^2 (-)
mpg-C_3N_4	$\ln(c_0/c) = 0.017\ t$	0.017	40.8	0.925
Ag1@mpg-C_3N_4	$\ln(c_0/c) = 0.058\ t$	0.058	12.0	0.990
Ag2@mpg-C_3N_4	$\ln(c_0/c) = 0.070\ t$	0.070	9.9	0.992
Cu1@mpg-C_3N_4	$\ln(c_0/c) = 0.115\ t$	0.115	6.0	0.995
Cu2@mpg-C_3N_4	$\ln(c_0/c) = 0.163\ t$	0.163	4.3	0.975

Table S8. Literature precedents for the degradation of emerging pharmaceutical contaminants using similar carbon nitride materials.

Catalyst	Contaminant	c_0 (mg L^{-1})	Light source	k (min^{-1})	Ref. [a]
Cu2@mpg-C_3N_4	Gemfibrozil	100	λ = 450 nm	0.163	This work
Co-pCN[1]	Oxytetracycline	20	300 W, λ > 420 nm	0.038	[1]
Ag-g-C_3N_4[2]	Naproxen	200	150 ± 5 mW/cm^2, $\lambda \geq$ 400 nm	0.192	[2]
Fe-g-C_3N_4[3]	Diclofenac	5	150 ± 5 mW/cm^2, vis	0.069	[3]
Co-OCN[4]	Bisphenol A	15	500 W, λ > 420 nm	0.029	[4]
K-CN[5]	RhB	100	250 W, 400 nm < λ < 800 nm	0.011	[5]
AKCN-Fe[6]	RhB	10	500 W, λ > 420 nm	0.018	[6]

[a] References for this table are reported at page S20 of the Supporting Information file.

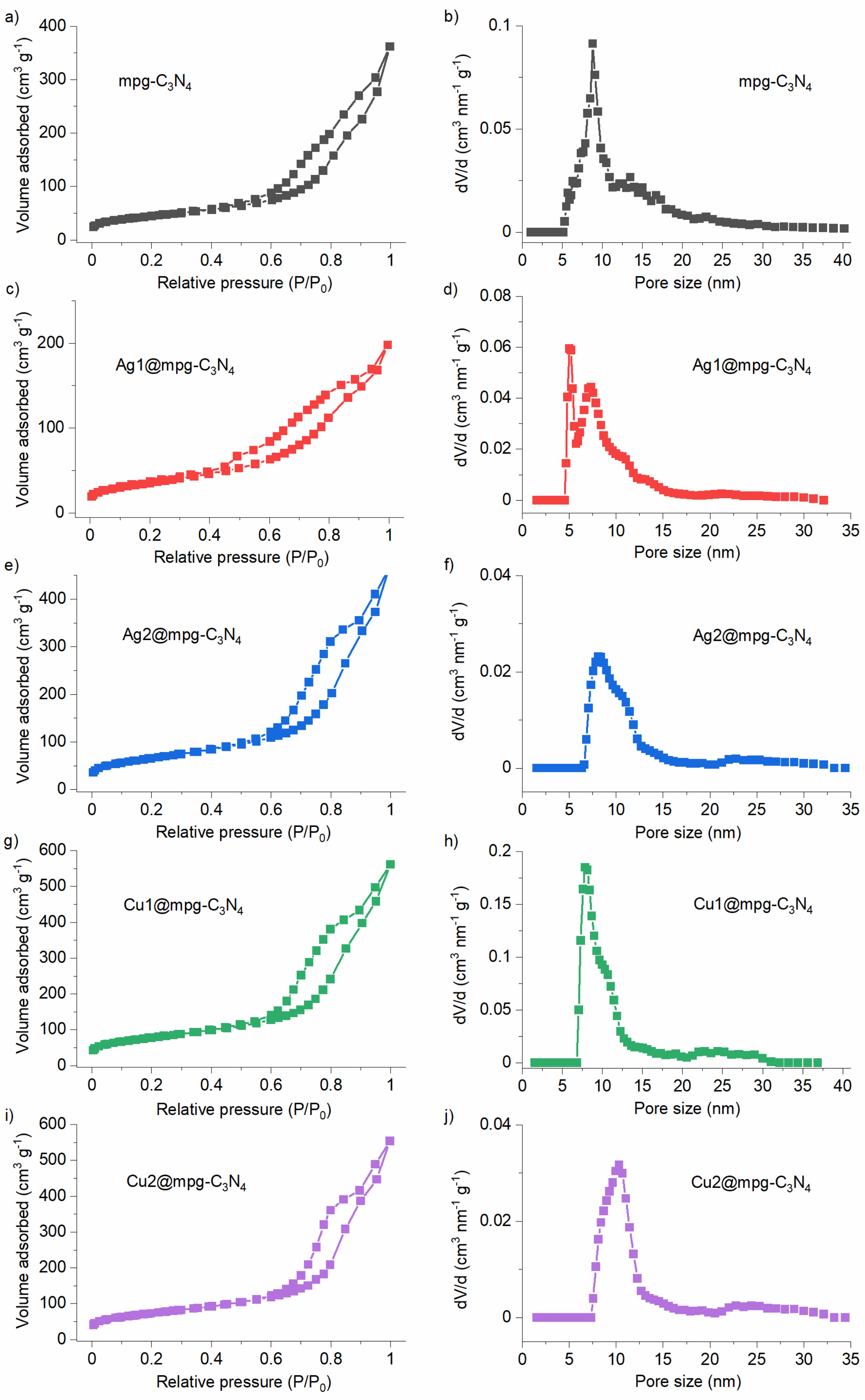

Figure S1. Nitrogen sorption isotherms at 77K of samples (a, c, e, g, i) and corresponding pore size distribution (b, d, f, h, j) calculated by a QSDFT model assuming cylindrical-shaped pores of samples.

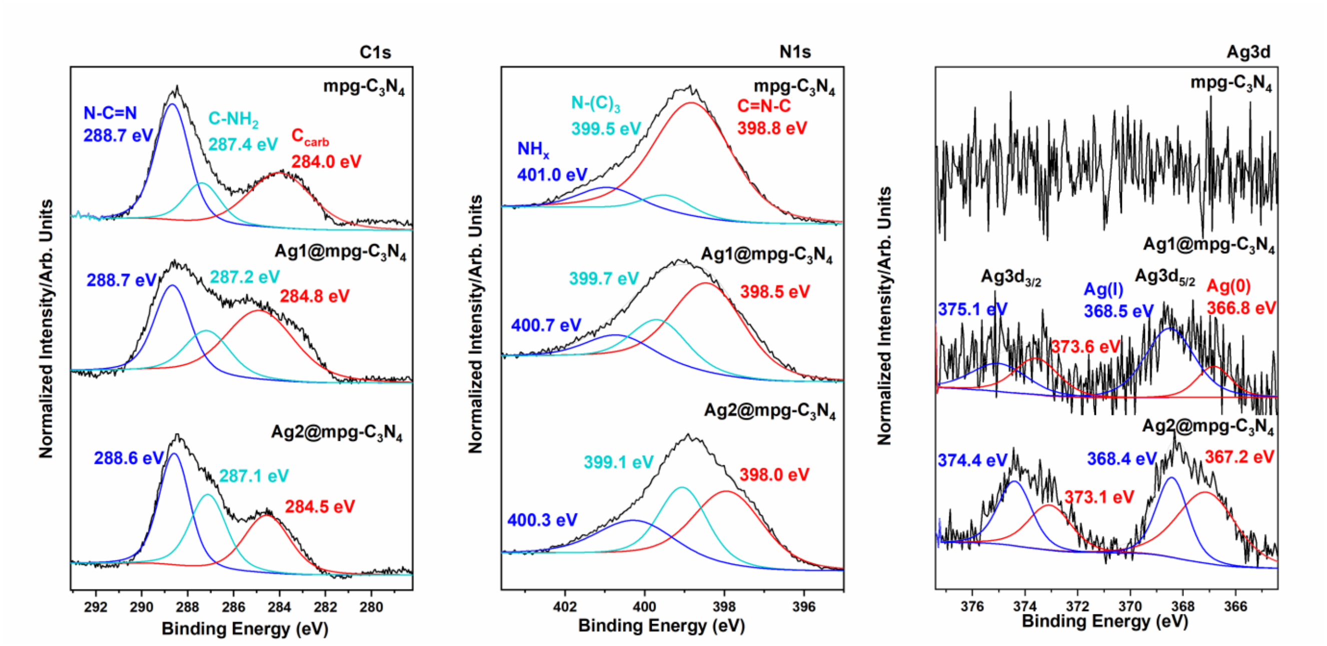

Figure S2. C 1*s*, N 1*s*, and Ag 3*d* X-ray photoelectron spectroscopy of mpg-C_3N_4, Ag1@mpg-C_3N_4, and Ag2@mpg-C_3N_4.

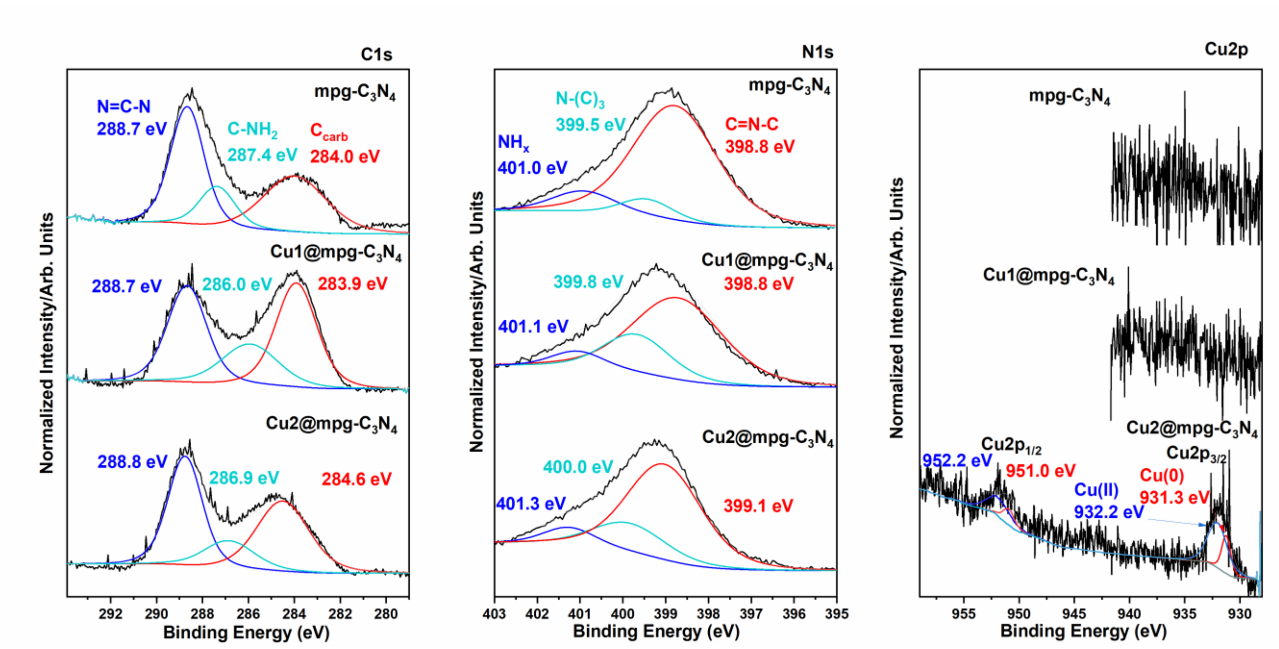

Figure S3. C 1*s*, N 1*s*, and Cu 2*p* X-ray photoelectron spectroscopy of mpg-C_3N_4, Cu1@mpg-C_3N_4, and Cu2@mpg-C_3N_4.

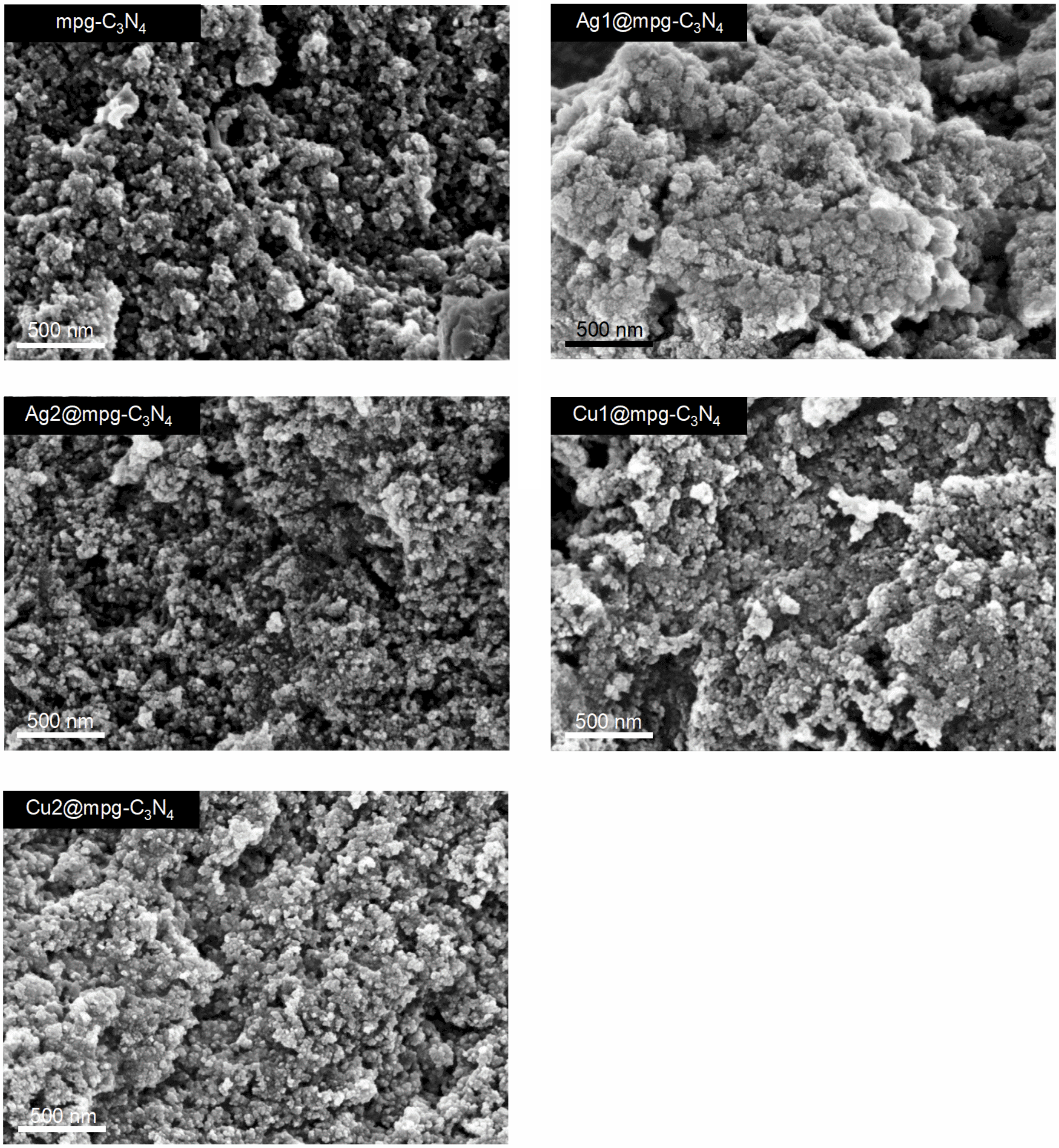

Figure S4. Scanning electron microscopy of the catalysts.

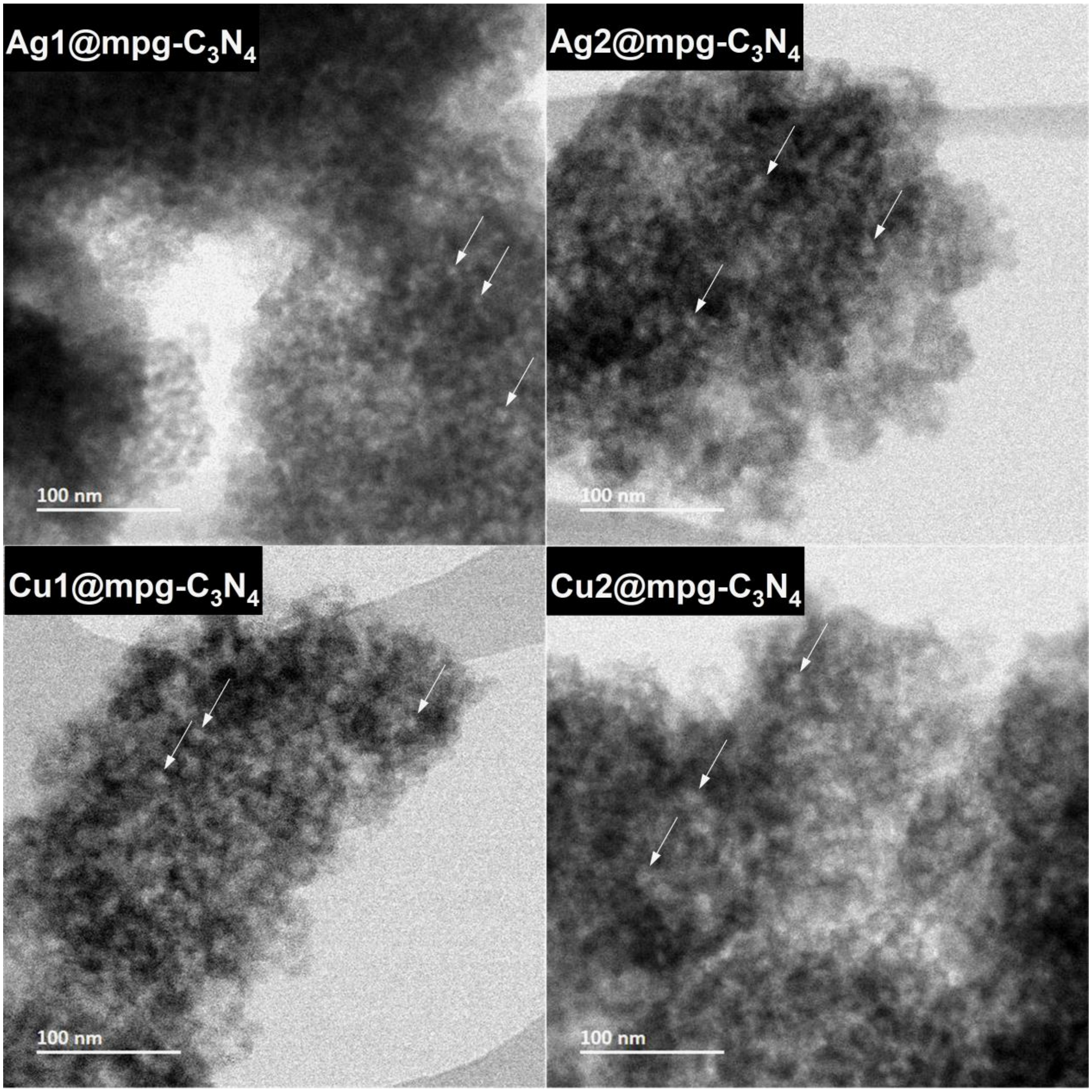

Figure S5. High-resolution transmission electron microscopy of the single-atom catalysts. Mesopores (bright spots) are highlighted with arrows and are well-distributed throughout the samples. Electron beam damage dominated by radiolysis, is one of the major challenges in high-resolution characterization in polymer science.[7] Carbon nitride materials are easily damaged upon exposure to the electron beam, which also induce moving of the sample during the observation and results in TEM images that are not well focused. Despite this, the mesoporous structure of the photocatalysts can be supported by Figure S5.

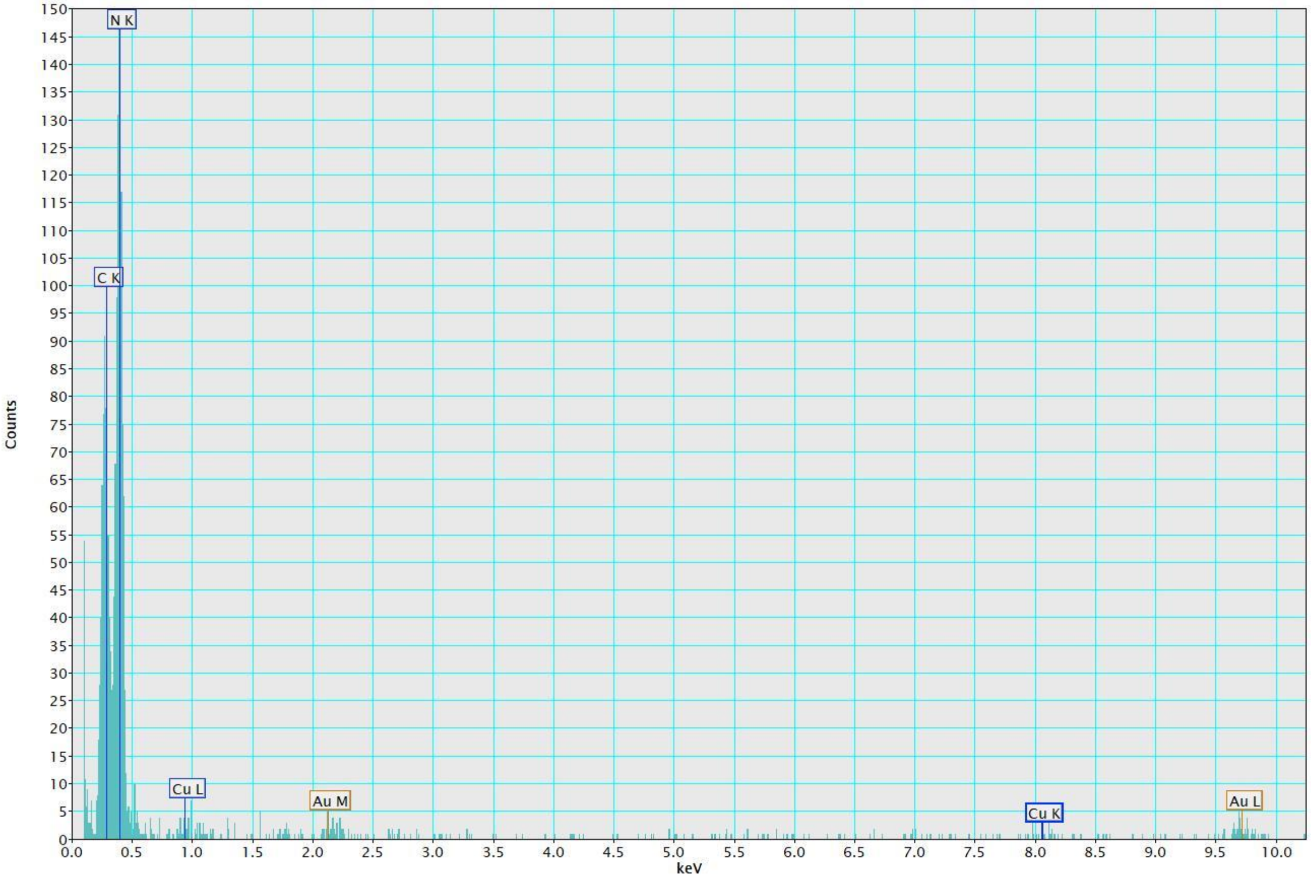

Figure S6. TEM-EDX spectrum of Cu2@mpg-C_3N_4. The Au peak is due to the material of the TEM grid.

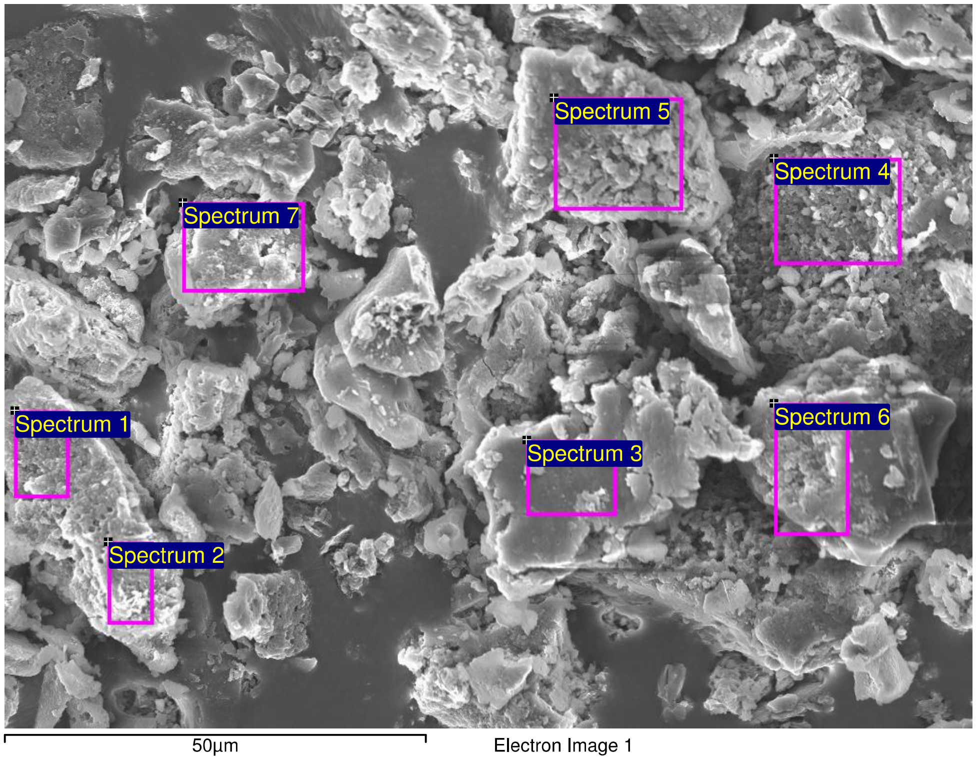

Processing option : All elements analysed (Normalised)

Spectrum	In stats.	C	N	O	Na	Si	Total
Spectrum 1	Yes	48.85	43.01	5.28	0.23	2.63	100.00
Spectrum 2	Yes	44.55	48.52	5.31	0.32	1.30	100.00
Spectrum 3	Yes	38.35	57.18	3.93	0.27	0.26	100.00
Spectrum 4	Yes	47.12	48.29	3.60	0.68	0.32	100.00
Spectrum 5	Yes	40.94	53.43	4.61	0.57	0.44	100.00
Spectrum 6	Yes	40.60	54.24	4.52	0.11	0.53	100.00
Spectrum 7	Yes	37.04	58.13	4.21	0.31	0.31	100.00
Mean		42.49	51.83	4.49	0.36	0.83	100.00
Std. deviation		4.45	5.45	0.64	0.20	0.87	
Max.		48.85	58.13	5.31	0.68	2.63	
Min.		37.04	43.01	3.60	0.11	0.26	

All results in weight%

Figure S7. SEM-EDX elemental analysis of mpg-C_3N_4. Na and O derive from washing the material with NH_4HF_2, Si derives from the residual of the template.

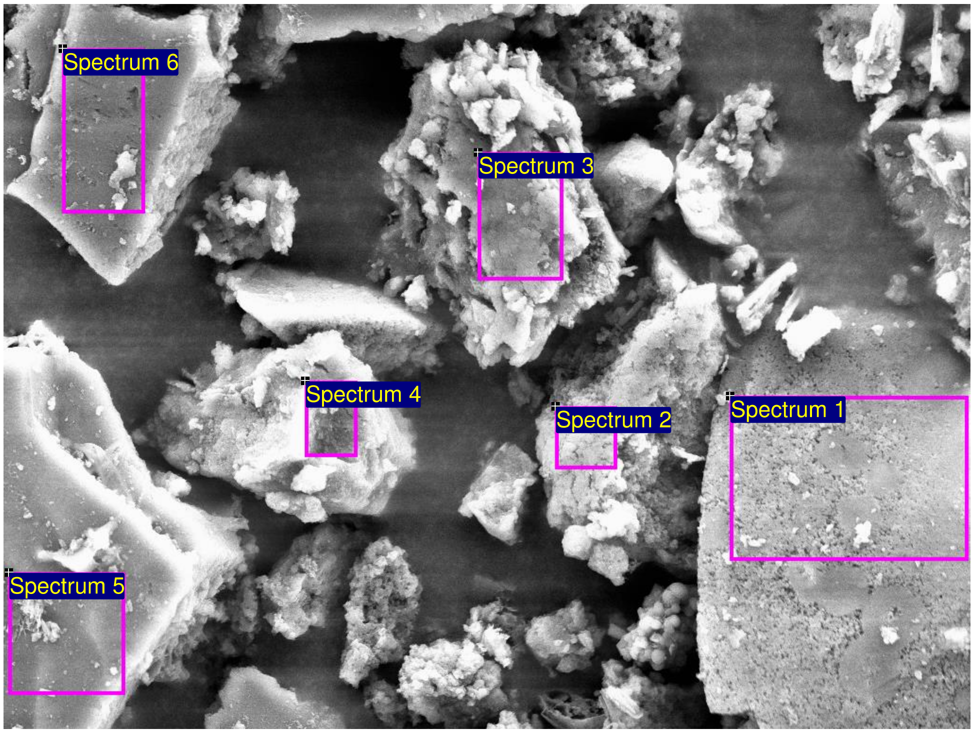

Processing option : All elements analysed (Normalised)

Spectrum	In stats.	C	N	O	F	Na	Si	Ag	Total
Spectrum 1	Yes	39.47	51.86	7.63		0.17	0.24	0.62	100.00
Spectrum 2	Yes	46.81	45.40	6.84		0.13	0.44	0.37	100.00
Spectrum 3	Yes	36.25	57.13	5.33		0.30	0.40	0.58	100.00
Spectrum 4	Yes	38.41	50.91	5.24	2.27	1.06	0.84	1.27	100.00
Spectrum 5	Yes	50.51	40.84	6.96		0.12	1.06	0.52	100.00
Spectrum 6	Yes	54.81	36.81	7.14		0.06	0.78	0.40	100.00
Max.		54.81	57.13	7.63	2.27	1.06	1.06	1.27	
Min.		36.25	36.81	5.24	2.27	0.06	0.24	0.37	

All results in weight%

Figure S8. SEM-EDX elemental analysis of Ag1@mpg-C_3N_4. Na, O and F derive from washing the material with NH_4HF_2, Si derives from the residual of the template.

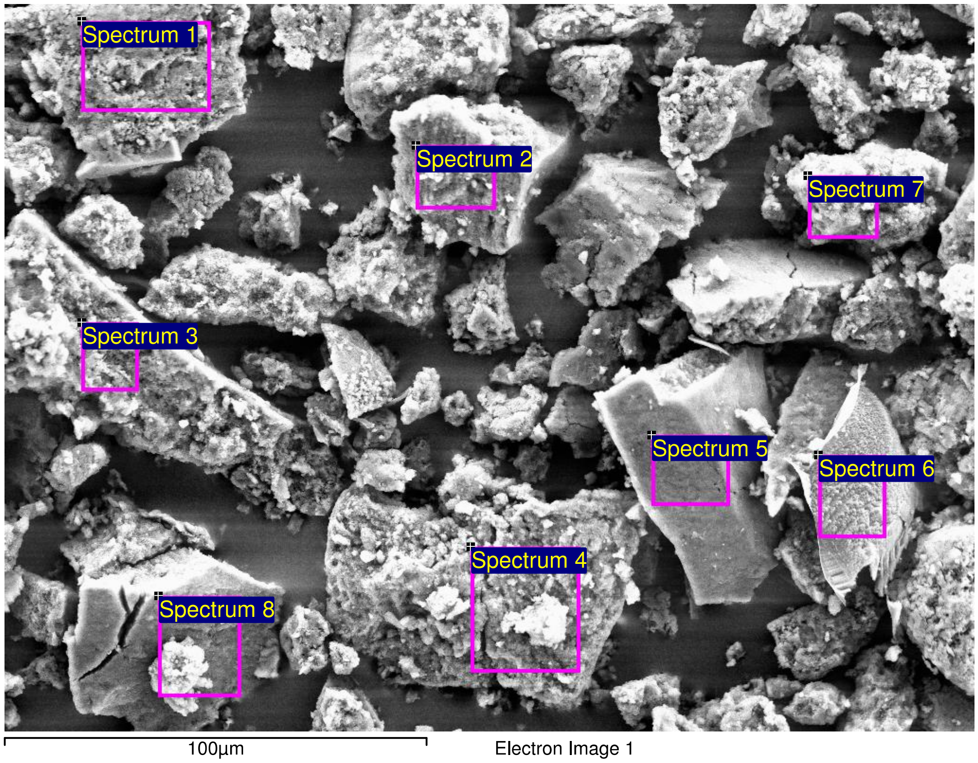

Processing option : All elements analysed (Normalised)

Spectrum	In stats.	C	N	O	Na	Si	Ag	Total
Spectrum 1	Yes	45.35	47.06	5.87	0.19	0.17	1.37	100.00
Spectrum 2	Yes	49.16	41.74	5.56	0.12	0.32	3.10	100.00
Spectrum 3	Yes	44.05	49.22	5.36	0.09	0.08	1.19	100.00
Spectrum 4	Yes	41.22	49.91	6.37	0.06	0.09	2.36	100.00
Spectrum 5	Yes	49.51	41.24	6.56	0.05	0.32	2.33	100.00
Spectrum 6	Yes	45.05	46.17	7.51	0.42	0.05	0.80	100.00
Spectrum 7	Yes	47.81	44.27	5.89	0.17	0.27	1.59	100.00
Spectrum 8	Yes	46.52	44.21	7.27	0.06	0.25	1.69	100.00
Mean		46.08	45.48	6.30	0.14	0.19	1.80	100.00
Std. deviation		2.77	3.20	0.78	0.12	0.11	0.75	
Max.		49.51	49.91	7.51	0.42	0.32	3.10	
Min.		41.22	41.24	5.36	0.05	0.05	0.80	

All results in weight%

Figure S9. SEM-EDX elemental analysis of Ag2@mpg-C_3N_4. Na, O and F derive from washing the material with NH_4HF_2, Si derives from the residual of the template.

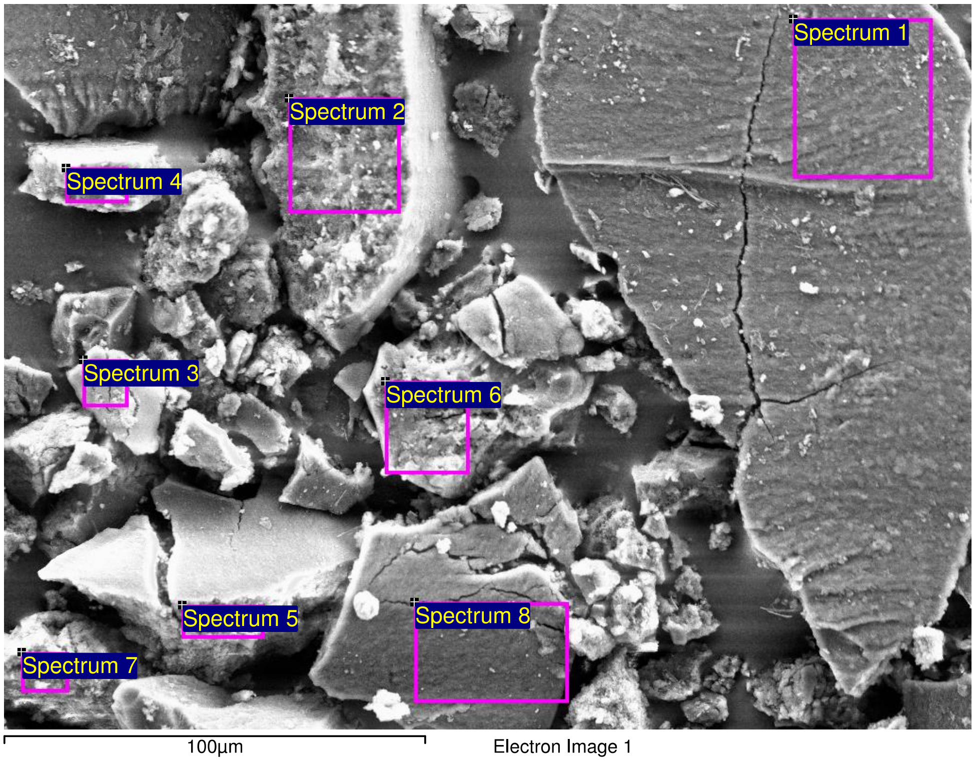

Processing option : All elements analysed (Normalised)

Spectrum	In stats.	C	N	O	Na	Si	Cu	Total
Spectrum 1	Yes	41.88	52.63	4.77	0.08	0.13	0.52	100.00
Spectrum 2	Yes	43.17	51.85	4.34	0.16	0.16	0.33	100.00
Spectrum 3	Yes	40.83	53.89	4.01	0.13	0.61	0.54	100.00
Spectrum 4	Yes	49.74	45.14	3.61	0.30	0.90	0.32	100.00
Spectrum 5	Yes	47.44	48.52	3.15	0.20	0.37	0.32	100.00
Spectrum 6	Yes	41.82	53.64	3.96	0.18	0.19	0.21	100.00
Spectrum 7	Yes	47.47	48.27	3.36	0.10	0.41	0.38	100.00
Spectrum 8	Yes	47.76	47.21	4.25	0.09	0.28	0.41	100.00
Mean		45.01	50.14	3.93	0.15	0.38	0.38	100.00
Std. deviation		3.44	3.28	0.54	0.07	0.26	0.11	
Max.		49.74	53.89	4.77	0.30	0.90	0.54	
Min.		40.83	45.14	3.15	0.08	0.13	0.21	

All results in weight%

Figure S10. SEM-EDX elemental analysis of Cu1@mpg-C_3N_4. Na, O and F derive from washing the material with NH_4HF_2, Si derives from the residual of the template.

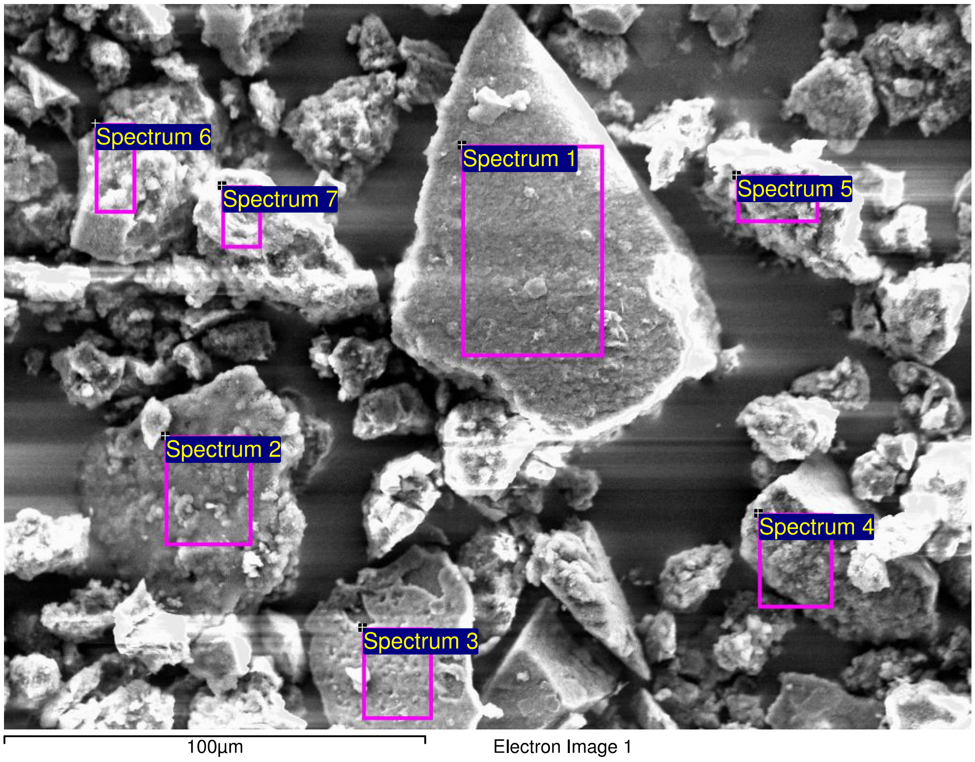

Processing option : All elements analysed (Normalised)

Spectrum	In stats.	C	N	O	Na	Si	Cu	Total
Spectrum 1	Yes	41.01	52.46	4.79	0.18	0.16	1.40	100.00
Spectrum 2	Yes	45.12	47.02	4.79	0.12	0.38	2.57	100.00
Spectrum 3	Yes	32.44	58.69	4.93	1.16	0.04	2.74	100.00
Spectrum 4	Yes	46.36	47.24	4.05	0.21	0.56	1.58	100.00
Spectrum 5	Yes	46.75	46.61	4.25	0.17	0.60	1.62	100.00
Spectrum 6	Yes	39.42	53.62	4.95	0.12	0.32	1.58	100.00
Spectrum 7	Yes	38.51	55.73	3.82	0.19	0.24	1.52	100.00
Mean		41.37	51.62	4.51	0.31	0.33	1.86	100.00
Std. deviation		5.16	4.78	0.46	0.38	0.20	0.55	
Max.		46.75	58.69	4.95	1.16	0.60	2.74	
Min.		32.44	46.61	3.82	0.12	0.04	1.40	

All results in weight%

Figure S11. SEM-EDX elemental analysis of Cu2@mpg-C_3N_4. Na, O and F derive from washing the material with NH_4HF_2, Si derives from the residual of the template.

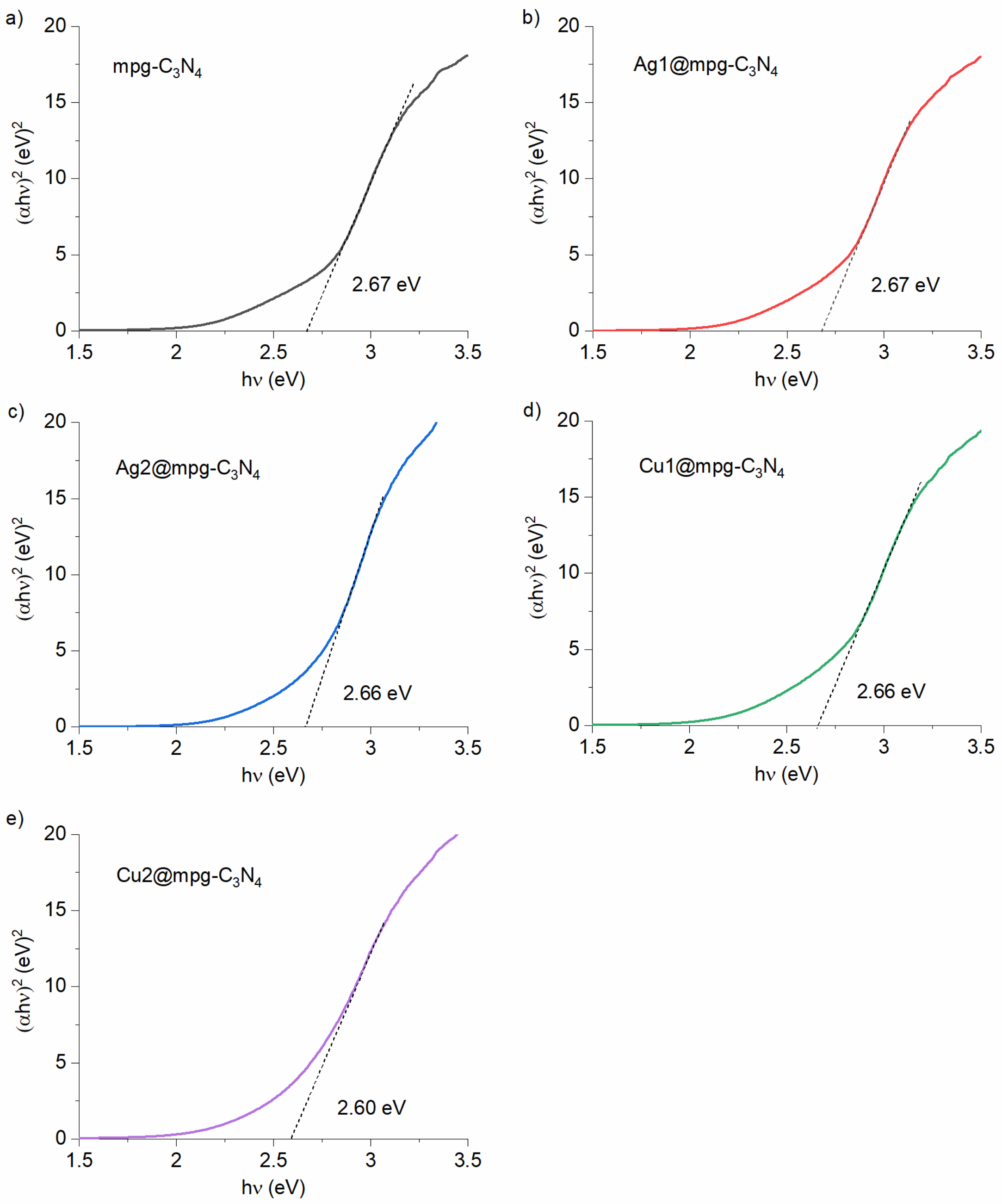

Figure S12. Tauc plots for the materials.

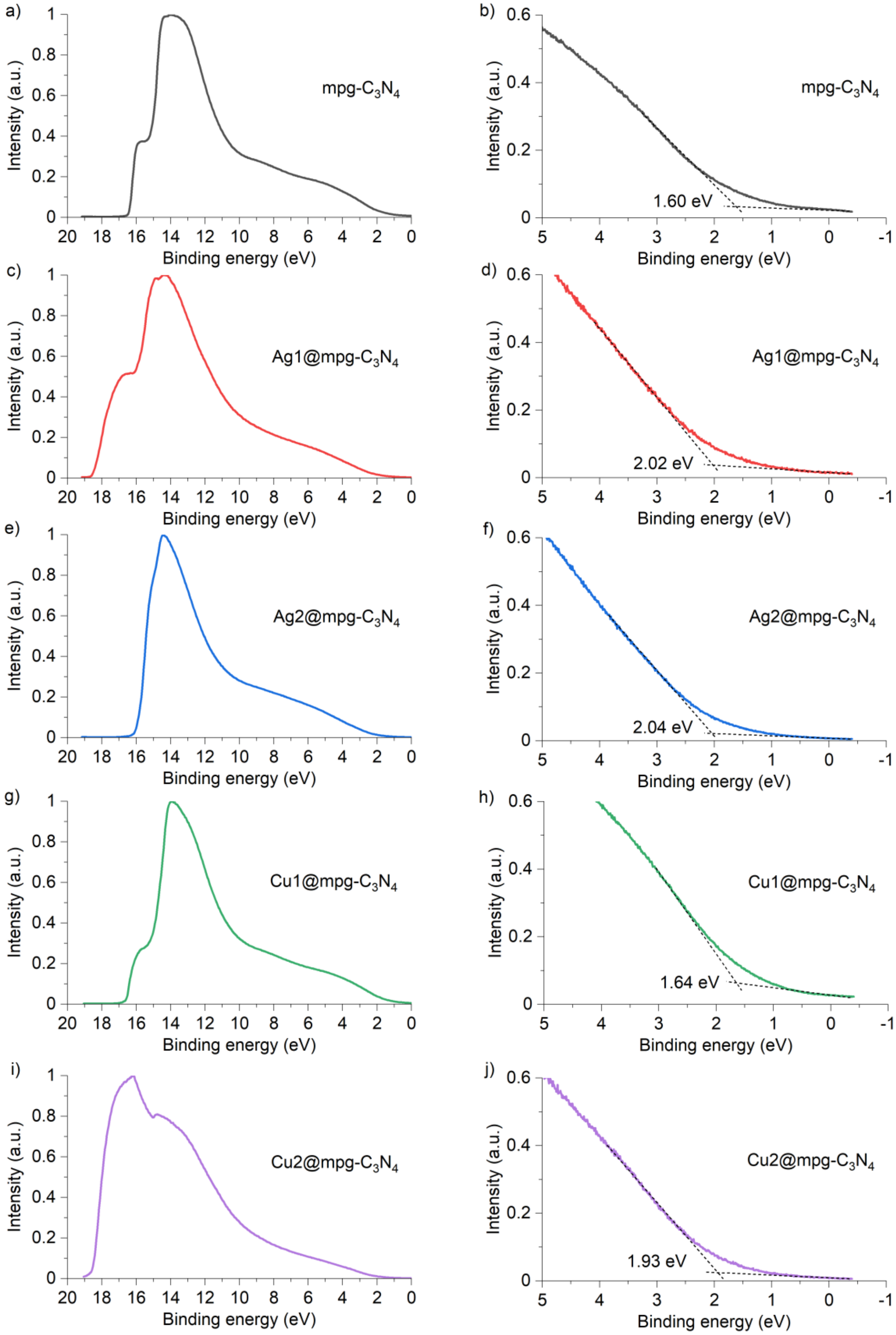

Figure S13. a, c, e, g, i) UPS spectra of the materials. b, d, f, h, j) Extended UPS spectra of the materials.

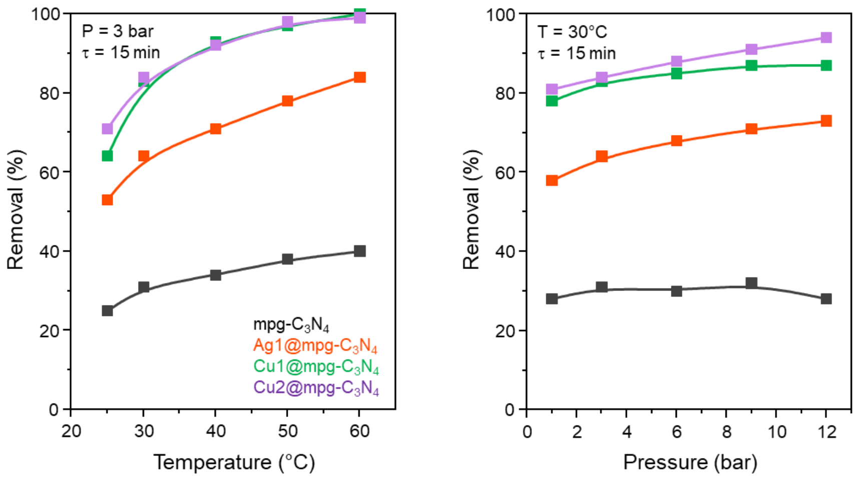

Figure S14. Effect of temperature and pressure on the degradation of Gemfibrozil in water over mpg-C_3N_4 (black), Ag1@mpg-C_3N_4 (red), Cu1@mpg-C_3N_4 (blue), and Cu2@mpg-C_3N_4 (green). Conditions: [Gemfibrozil] = 100 ppm, m_{cat} = 100 mg, light wavelength = 450 nm.

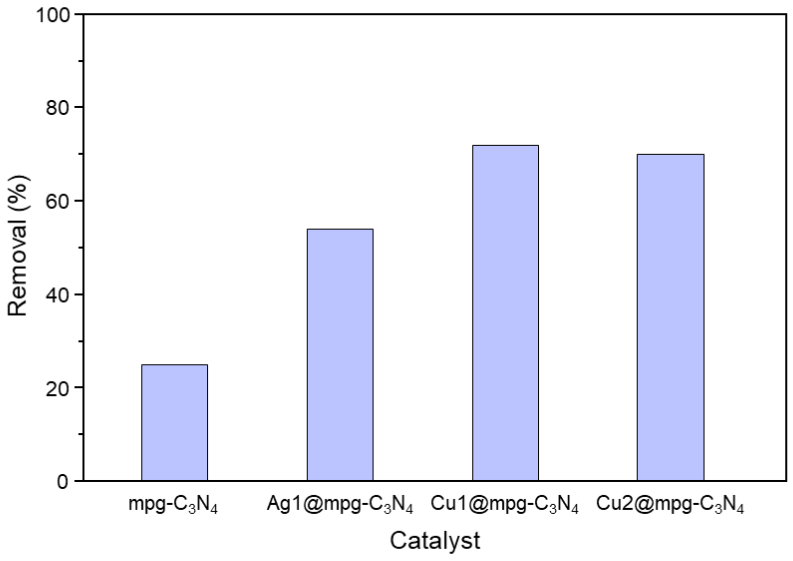

Figure S15. Removal of Gemfibrozil in water over different photocatalysts under batch-type conditions, at [Gemfibrozil] = 100 ppm, m_{cat} = 100 mg, temperature = 30°C, pressure = 3 bar, reaction time = 15 min, and light wavelength = 450 nm. The figure demonstrates that the reactor geometry does not substantially alter the order of reactivity of the materials, with the two Cu-based single-atom catalysts remaining the most active.

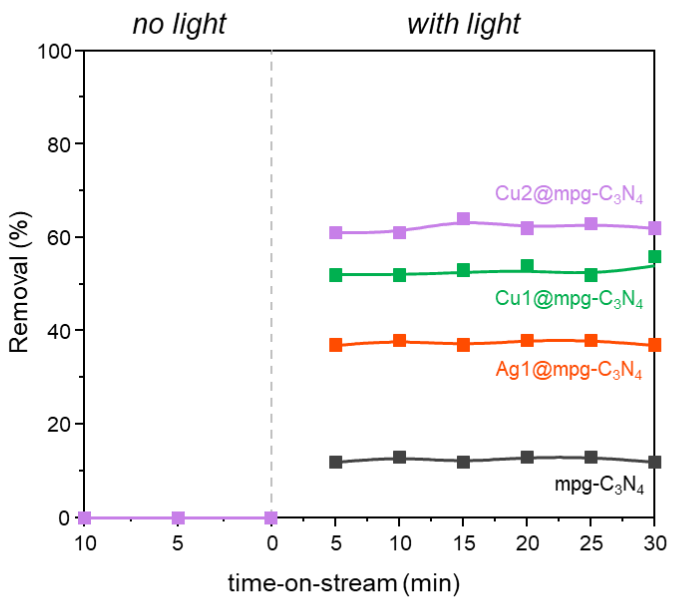

Figure S16. Catalyst performance as a function of the time-on-stream, at [Gemfibrozil] = 100 ppm, m_{cat} = 100 mg, temperature = 30°C, pressure = 3 bar, and residence time *rt* = 5 min. The figure highlights the absence of Gemfibrozil degradation in the absence of light irradiation, and the stable photocatalytic performance of the materials over an extended period. All data were collected under continuous-flow conditions.

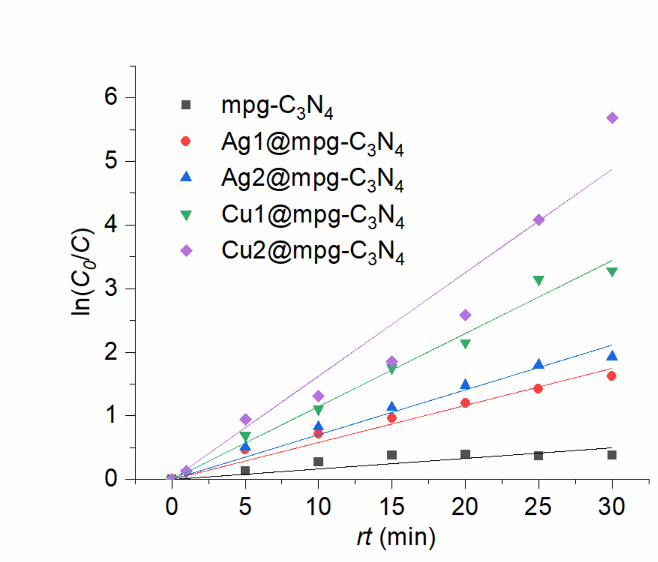

Figure S17. Pseudo-first-order kinetic fitting curves. The analysis refers to the results in Figure 4a in the main manuscript. Conditions: [Gemfibrozil] = 100 ppm, m_{cat} = 100 mg, temperature = 30°C, pressure = 3 bar, light wavelength = 450 nm.

References

[1] Yang, Y.; Zeng, G.; Huang, D.; Zhang, C.; He, D.; Zhou, C.; Wang, W.; Xiong, W.; Song, B.; Yi, H.; Ye, S.; Ren, X. In Situ Grown Single-Atom Cobalt on Polymeric Carbon Nitride with Bidentate Ligand for Efficient Photocatalytic Degradation of Refractory Antibiotics. *Small* **2020**, *16*, 2001634.

[2] Zhao, Z.; Zhang, W.; Liu, W.; Li, Y.; Ye, J.; Liang, J.; Tong, M. Single-Atom Silver Induced Amorphization of Hollow Tubular g-C_3N_4 for Enhanced Visible Light-Driven Photocatalytic Degradation of Naproxen. *Sci. Total. Environ.* **2020**, *742*, 140642.

[3] Zhao, Z.; Zhang, W.; Liu, W.; Li, Y.; Ye, J.; Liang, J.; Tong, M. Activation of Sulfite by Single-Atom Fe Deposited Graphitic Carbon Nitride for Diclofenac Removal: The Synergetic Effect of Transition Metal and Photocatalysis. *Chem. Eng. J.* **2021**, *407*, 127167.

[4] Yu, Y.; Wu, S.; Gu, J.; Liu, R.; Wang, Z.; Chen, H.; Jiang, F. Visible-Light Photocatalytic Degradation of bisphenol A Using Cobalt-To-Oxygen Doped Graphitic Carbon Nitride with Nitrogen Vacancies via Metal-To-Ligand Charge Transfer. *J. Hazard. Mater.* **2020**, *384*, 121247.

[5] Hu, S.; Li, F.; Fan, Z.; Wang, F.; Zhao, Y.; Lv, Z. Band Gap-Tunable Potassium Doped Graphitic Carbon Nitride with Enhanced Mineralization Ability. *Dalton Trans* **2015**, *44*, 1084-1092.

[6] Gao, Y.; Duan, J.; Zhai, X.; Guan, F.; Wang, X.; Zhang, J.; Hou, B. Photocatalytic Degradation and Antibacterial Properties of Fe^{3+}-Doped Alkalized Carbon Nitride. *Nanomaterials* **2020**, *10*, 1751.

[7] Egerton, R. F.; Li, P.; Malac, M. Radiation Damage in the TEM and SEM. *Micron* **2004**, *35*, 399-409.
